# A multi-object deep neural network architecture to detect prostate anatomy in T2-weighted MRI: Performance evaluation

**DOI:** 10.3389/fnume.2022.1083245

**Published:** 2023-02-06

**Authors:** Maria Baldeon-Calisto, Zhouping Wei, Shatha Abudalou, Yasin Yilmaz, Kenneth Gage, Julio Pow-Sang, Yoganand Balagurunathan

**Affiliations:** ^1^ Departamento de Ingeniería Industrial and Instituto de Innovación en Productividad y Logística CATENA-USFQ, Universidad San Francisco de Quito, Quito, Ecuador; ^2^ Department of Machine Learning, H. Lee Moffitt Cancer Center and Research Institute, Tampa, FL, United States; ^3^ Department of Electrical Engineering, University of South Florida, Tampa, FL, United States; ^4^ Diagnostic Radiology, H. Lee Moffitt Cancer Center and Research Institute, Tampa, FL, United States; ^5^ Genitourinary Oncology, H. Lee Moffitt Cancer Center and Research Institute, Tampa, FL, United States

**Keywords:** prostate cancer, prostate segmentation, machine learning, deep learning, neural network, neural architecture search, EMONAS, AdaEn-Net

## Abstract

Prostate gland segmentation is the primary step to estimate gland volume, which aids in the prostate disease management. In this study, we present a 2D-3D convolutional neural network (CNN) ensemble that automatically segments the whole prostate gland along with the peripheral zone (PZ) (PPZ-SegNet) using a T2-weighted sequence (T2W) of Magnetic Resonance Imaging (MRI). The study used 4 different public data sets organized as Train #1 and Test #1 (independently derived from the same cohort), Test #2, Test #3 and Test #4. The prostate gland and the peripheral zone (PZ) anatomy were manually delineated with consensus read by a radiologist, except for Test #4 cohorts that had pre-marked glandular anatomy. A Bayesian hyperparameter optimization method was applied to construct the network model (PPZ-SegNet) with a training cohort (Train #1, *n* = 150) using a five-fold cross validation. The model evaluation was performed on an independent cohort of 283 T2W MRI prostate cases (Test #1 to #4) without any additional tuning. The data cohorts were derived from The Cancer Imaging Archives (TCIA): PROSTATEx Challenge, Prostatectomy, Repeatability studies and PROMISE12-Challenge. The segmentation performance was evaluated by computing the Dice similarity coefficient and Hausdorff distance between the estimated-deep-network identified regions and the radiologist-drawn annotations. The deep network architecture was able to segment the prostate gland anatomy with an average Dice score of 0.86 in Test #1 (*n* = 192), 0.79 in Test #2 (*n* = 26), 0.81 in Test #3 (*n* = 15), and 0.62 in Test #4 (*n* = 50). We also found the Dice coefficient improved with larger prostate volumes in 3 of the 4 test cohorts. The variation of the Dice scores from different cohorts of test images suggests the necessity of more diverse models that are inclusive of dependencies such as the gland sizes and others, which will enable us to develop a universal network for prostate and PZ segmentation. Our training and evaluation code can be accessed through the link: https://github.com/mariabaldeon/PPZ-SegNet.git.

## Introduction

Prostate carcinoma is the second most frequent cancer in men, accounting for 3.8% of male fatalities globally and a primary cause of death in over 48 countries (1, 2). Multi-parametric magnetic resonance imaging (mpMRI) is used to visualize and quantify the tissue using per- fusion/permeability characteristics, non-invasively which helps diagnose, stage, monitor and evaluate the prostate cancers (3–5). Prostate segmentation is also frequently applied in various routine clinical practices such as radiation therapy planning (6, 7), MRI-ultrasound image-guided biopsy (8, 9), as well as focal therapy (10). A manual delineation is often used in clinical practice, which is a laborious task with poor reproducibility and shown to have a high inter-observer variation (11), subjective to expert training as recently reported. To improve the time-consuming nature of manual prostate delineation, current PI-RADS (Prostate Imaging-Reporting and Data System) guidelines recommended using a simpler geometric shape such as an ellipsoid for fast estimation of the prostate volume.

In the past, there were many methods proposed for automated prostate segmentation which started with atlas-based segmentation (12), deformable models (13), machine learning based methods on marginal space learning (14), and c-means clustering with zonal morphology (15). One additional successful attempt was to use pattern recognition methods to delineate glandular architecture (16). Currently, deep learning (DL) has shown tremendous promise in modeling complex problems in oncology (17). It has also been widely applied to segment various anatomical structures across different modalities (18, 19). Particularly, deep convolutional neural networks (CNNs) have achieved great success by automatically learning to extract the most important features for image characterization (18, 20). CNN architectures are usually composed of multiple layers, in which the initial layers extract local information and low-level features, while the deeper layers learn to recognize more complex objects (21). These networks have shown to surpass human performance on some tasks (22). For example, CheXNet achieved a better performance in the detection of pneumonia from chest x-rays than the average performance of four radiologists (23, 24). Recently, the Encoder-Decode based CNN architectures (U-Net) have seen enormous adoption in segmentation tasks due to its ability to adapt to every new dataset (25).

CNNs for medical image segmentation are usually divided into 2D or 3D networks based on how they handle volumetric data. 2D networks segment the anatomical structures in a slice-wise manner and then concatenate the results in the *z*-axis. These architectures are very good at extracting intra-slice information, computationally efficient, and capture long-range pixel relationships while keeping the input size reasonable. However, volumetric information is not considered during inference. By contrast, 3D networks directly process the volumetric input, being able to consider both intra- and inter-slice information during prediction. Nevertheless, they are computationally expensive and 3D networks have shown to provide worse performance than 2D networks when there is high intra-slice resolution (26).

Most machine learning/deep network models assume the data used for training and testing are independent and identically distributed with samples from a reference probability distribution, which can pose a certain level of limitation on the model's generalization. It is well noted that the performance of a model usually degrades when tested on a distinct dataset due to the domain shift (27, 28). Moreover, it is well recognized that medical image datasets are most often heterogeneous due to scan, acquisition protocol, and subject level differences. Therefore, it becomes indispensable to develop networks which can be transferred between datasets without a substantial performance drop (29) or a need for additional training or tuning (30, 31).

In this work, we propose a multi-object deep CNN ensemble, modified from our previously published model (32), which applies Network Architecture Search (33) to segment multiple anatomical regions in the prostate. We refer to the model as the **P**rostate gland and **P**eripheral **Z**one **Seg**mentation **Net**work (PPZ-SegNet). The PPZ-SegNet is composed of a two-path 2D and 3D CNN, which are automatically constructed using a Bayesian hyperparameter optimization method. As demonstrated in previous work (32, 34) using an ensemble of 2D and 3D CNNs allows the model to exploit intra-slice and inter-slice information. Moreover, the ensemble model improves generalization that allows the network to perform better across cohorts. PPZ-SegNet differs from prior work from our group (32) and others (35) in terms of the problem being addressed, segmentation task, neural network architecture, hyperparameters optimized, and the optimization method applied. Particularly, an adaptive ensemble was proposed (32) for medical image segmentation that applies a multi-objective evolutionary based algorithm to construct efficient and accurate networks. In this work, the focus is on analyzing the effect that distinct training and testing cohorts have in the performance of a segmentation network. Moreover, PPZ-SegNet is trained on the task of prostate and peripheral zone (PZ) segmentation. To achieve the latter, the architecture is modified to include 2 decoder paths. One decoder produces the prostate segmentation, while the other predicts the PZ segmentation. Moreover, the hyperparameters being optimized during construction include hyperparameters pertinent to this new architecture and used during the ensemble training. Finally, a Bayesian optimization method is implemented to maximize the segmentation accuracy.

The study used 4 different public cohorts available on the Cancer Imaging Archive (TCIA). The prostate gland and zonal anatomy on the patient scans were contoured by our trained clinical experts, except for one of the cohorts that came with regional annotations. We find that our optimized PPZ-SegNet architecture shows promising performance in our training cohort. In this paper, we describe our PPZ-SegNet network and its hyperparameter optimization procedure, validating the trained network in a set of independent, diverse test cohorts to show promise in the use of optimized deep networks in oncology.

## Methods

### Datasets

The dataset used in our study contains 433 MRI-T2W images curated from 4 different open-source collections: PROSTATEx Challenge (Train #1, *n* = 150 & Test #1, *n* = 192), PROSTATE-MRI Prostatectomy (Test #2, *n* = 26), QIN-PROSTATE-Repeatability (Test #3, *n* = 15), MICCAI PROMISE12-Challenge (Test #4, *n* = 50). Patients image scans for the above cohorts are available on the TCIA website (https://www.cancerimagingarchive.net/) under the collection titled: “PROSTATEx”, “QIN-PROSTATE-Repeatability”, and “Prostate Fused-MRI-Pathology”. The PROMISE12 challenge data is available through the organizer's website: https://promise12.grand-challenge.org/. The patient scans in these cohorts were collected using different MRI scanners that broadly fall under these vendors, i.e., Siemens, Philips and GE Medical Systems, following their respectively institutional imaging protocols. The image scans were reviewed by an experienced clinical reader (radiologists with research/clinic roles for more than 5 years of clinical mpMRI reading experience) from the Moffitt Cancer Center (Tampa, FL, United States). The prostate and PZ regions were manually contoured using semi-automatic annotation tools in our research PACS (MIM Software Inc., Cleveland, OH, United States) based on the axial views of the MRI-T2W image. The manual reference contours were made on all MRI-T2W images except those from PROMISE12 dataset, which came with the annotated prostate glandular structures (no PZ). The prostate and PZ contours as well as the MRI-T2W anatomy images were exported from our research PACS as DICOM/RT [radiotherapy] images.

### Preprocessing of image data

The images included in the dataset exhibit variation in resolution and size. Specifically, the in-plane resolution varies from 0.2 to 0.65 mm^2^, while the slice thickness (through-plane) resolution ranges from 2 to 4.5 mm for patients across the cohorts. The images were pre-processed by resampling them to a 0.5 × 0.5 × 3 mm^3^ spatial resolution using B-spline interpolation and resizing to a standard reference plane (256 × 256 × 23). Furthermore, the pixel intensities are clipped within 3 standard deviations from the mean and rescaled to an interval of [0,1] in a slice-wise manner.

The manual contours were annotated using MIM software on the original MRI-T2W images and saved as RT structures [radiation therapy format], which are 2D polygons slice-by-slice. The vertices of these 2D polygons are saved in the DICOM header file under the Contour Data tag (3006,0050). The annotation images are transformed to the PATIENT coordinate system, which is derived using the vector cross product based on the information in the Image Orientation tag (0020,0037) and the Image Position tag (0020,0032). The above procedure was implemented using our in-house software written in MATLAB (version R2022a; MathWorks, Natick, MA, United States). Both the image volumes and annotations are saved in MATLAB Data format.

### The CNN architecture

**Figure 1:**
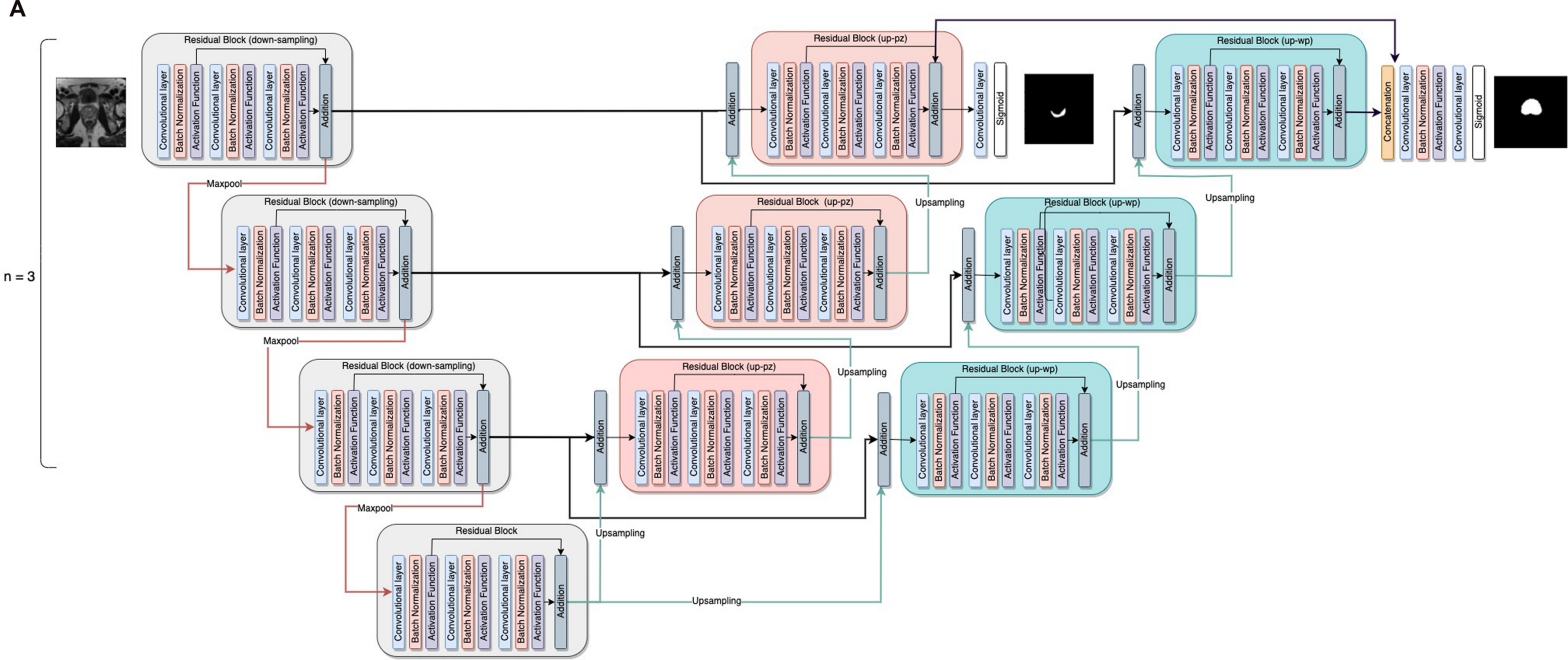
General structure of the PPZ-SegNet. (**A**) architecture is composed of a down-sampling path (gray residual blocks) and two up-sampling paths, denoted as up-pz (orange residual blocks) and up-wp (blue residual blocks). The up-pz path produces the peripheral zone segmentation, while the up-wp produces the whole prostate segmentation. (**B**) Three-step fitting process for the PPZ-SegNet. In step 1, the 2D CNN architecture is constructed using a Bayesian Optimization approach. In step 2, the 3D CNN architecture is constructed in a similar way as the 2D CNN. In step 3, the 2D CNN and 3D CNN are fully trained using a 5 fold-scheme. The final prediction is the majority voting of the five 2D-3D CNN ensembles.

The PPZ-SegNet neural architecture consists of a 2D CNN and 3D CNN. Both CNNs have a similar structure, the 2D CNN receives the pre-processed 2D slices of the prostate axial dataset (256 × 256 pixels) as input and applies 2D convolutions, while the 3D CNN is trained with pre-processed 3D cropped volumes (128 × 128 × 23 voxels) and uses 3D convolutions. The general structure of the networks is shown in Figure 1. The networks are composed of a down-sampling path followed by two up-sampling paths, denoted as up-pg (for prostate gland) and up-pz (for PZ). The down-sampling path receives as input the prostate image and through the application of convolutional and max-pooling operations extracts the most important image features for the segmentation task. The up-sampling paths, by contrast, receive the extracted features and through the application of up-sampling and convolutional operations increase the size of the feature maps until the segmentations of the prostate gland and PZ have been achieved through the up-pg and up-pz paths, respectively.

**Figure:**
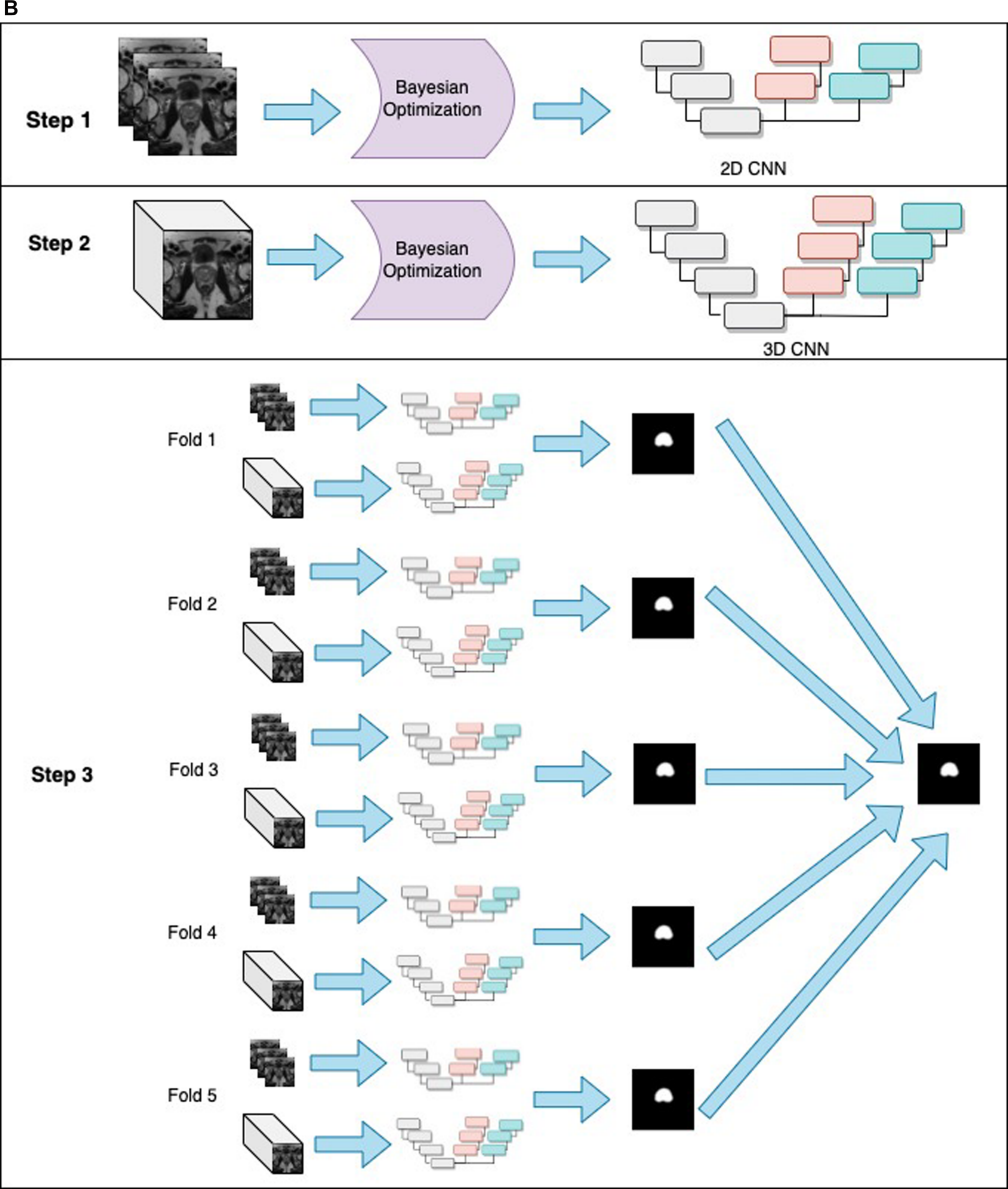


The down-sampling and up-sampling paths are composed of n residual blocks each, where the value of n is defined using the Bayesian optimization approach. The down-sampling and up-sampling paths are connected through a residual block in the middle, forming a U-shape architecture. Each residual block consists of 3 sequential convolutional blocks, in which a convolution block has a zero-padded convolutional layer, batch normalization layer, and an activation function layer. To prevent overfitting, a spatial dropout layer is included before the residual blocks, with exception of the first residual block. Furthermore, the features extracted from the last residual block of the up-pz path are concatenated with the features of the last residual block of the up-pg path, so that the information extracted about the PZ segmentation can be considered when predicting the segmentation of the prostate gland. The last convolutional layers of the up-pg path and up-pz path have a kernel window of size 1 and a sigmoid activation function.

Seven model hyperparameters have been left unset and will be optimized using Bayesian hyperparameter optimization. These hyperparameters correspond to the number of residual blocks in the down-sampling and up-sampling paths, number of filters on each residual block, activation function applied after the convolutional operation, kernel size for the 3 convolutional layers inside the residual blocks, and spatial-dropout probability. It is important to mention that all residual blocks have the same architecture, hence by defining the kernel size for the 3 convolutional layers, we are defining the kernel size for all the residual blocks. Moreover, the dropout probability is kept at the same level at the spatial dropout layers and a similar approach was taken to maintain the function at all the activation functional layers. During training, data augmentation is implemented in real time to all training images in an epoch to prevent overfitting. The magnitude of the five-data-augmentation operations is optimized using the same optimization approach. The five-data-augmentation operations implemented in the network model were rotation, width shift, height shift, zoom, and horizontal flip.

### Bayesian optimization approach

Selecting the optimal hyperparameter values for a neural network architecture is a non-linear optimization problem in which the decision variables correspond to the hyperparameters being optimized, and the objective is to minimize the error on the validation set. Let *N* denote the number of hyperparameters being optimized, and *Ω_j_* the search space of the *j*-th hyperparameter. Then, the overall hyperparameter search space is defined as *Ω* = *Ω*_1_ × *Ω*_2_ × *Ω*_3_ … *Ω_N_*. If *λ* refers to a vector of hyperparameter values, the hyperparameter optimization problem can be mathematically modeled as:

$$\lambda^{*}\;=\;\mathrm{argmi}n_{\lambda \in \Omega }L(D_{\mathrm{train}},\;D_{\mathrm{valid}}),$$

where *L*(*D*_train_,*D*_valid_) is the loss function that measures the error of the model with *λ* hyperparameter values trained on the *D*_train_ training set and evaluated on the *D*_valid_ validation set. Moreover, λ* denotes the optimal hyperparameter values that minimize the loss function. The training and validation set used for the Bayesian optimization in this study are composed of 150 cases from the PROSTATEx Challenge (referred to as Train #1).

In the present work, *λ* is a vector in which each component corresponds to one of the hyperparameters. In addition, the overall search space *Ω* is the cross-product of the search domains of each hyperparameter. Finally, the loss function *L*(*D*_train_,*D*_valid_) implemented is based on the Dice similarity coefficient as shown below and denominated as the Dice loss:

$$L(D_{\mathrm{train}},\;D_{\mathrm{valid}})=1-\frac{2\sum_{i}\hat{y_{i}}y_{i}}{\sum_{i}{\hat{y}}_{i}^{2}+\sum_{i}y_{i}^{2}};$$

where *y_i_* refers to the ground truth value of pixel *i*, and $\hat{y_{i}}$ the predicted probability for pixel *i*. The Dice coefficient measures the relative overlap between the predicted segmentation and ground truth segmentation; therefore, it is useful when there is an imbalance between background and foreground pixels. The Dice coefficient ranges between 0 and 1, where 1 denotes a perfectly predicted segmentation. Hence by minimizing “1 − *Dice coefficient*”, we are maximizing the segmentation accuracy.

Bayesian optimization is a sequential model-based approach characterized by a probabilistic surrogate model *f* and an acquisition function α. The probabilistic surrogate consists of a prior distribution that captures the belief behavior of the loss function $L(D_{\mathrm{train}},\;D_{\mathrm{valid}})$. In each iteration *t*, a new hyperparameter vector $\lambda_{t}$ is selected to construct a CNN and evaluated using a validation set. This new point is used to update the prior one into a posterior distribution. The posterior information is used by the acquisition function to decide which hyperparameter vectors to evaluate next. In this search, it is necessary to consider the criteria for exploration (sampling from areas with high uncertainty) and exploitation (sampling points with high values). The output of the algorithm is the hyperparameter vector $\lambda^{*}$ that optimizes the loss function. The Bayesian optimization algorithm applied to optimize the 12 hyperparameters that constructs the 2D CNN and 3D CNN is shown below (Algorithm 1).

**Algorithm 1:** Bayesian Optimization Algorithm.

Input:
- Number of iterations to run T - Acquisition function α - Probabilistic surrogate model f
Output:
- $\lambda^{*}$ optimal hyperparameter vector
Start loop: *t* = 1, 2,…, *T*:
1. Find $\lambda_{t}$ by optimizing the acquisition function α
$\lambda_{t}=\mathrm{argmin}\;\alpha (\lambda \;,\;D_{t})\;\;\;$
2. Train the CNN with the $\lambda_{t}$ hyperparameters and evaluate on the validation set. Calculate the loss function value $L_{t}(D_{\mathrm{train}},\;D_{\mathrm{valid}})$. 3. Augment the data $D_{t+1}=\{D_{t},(\;\lambda_{t},\;L_{t})\}$ 4. Update the posterior distribution of the probabilistic surrogate model *f* with $D_{t+1}$
End loop

### Ensemble formation

Once the architectures for the 2D CNN and 3D CNN are optimized, we form the PPZ-SegNet ensemble solely using Train #1 from the ProstateX dataset. First, Train #1 is divided into 5 folds, where 80% of the images are randomly assigned to the training set and 20% to the validation set. Each fold is used to fully train the 2D CNN and 3D CNN architectures. Then, the predictions from the 2D CNN and 3D CNN are combined by averaging their softmax probability maps. This creates a set of 5 2D–3D CNN ensembles that produces 5 predicted segmentations. Lastly, the final segmentation was obtained by aggregating the predicted segmentations using a majority voting scheme.

In the training phase, the 2D CNN was trained for 3,000 epochs, while the 3D CNN was trained for 2,000 epochs. The weights with the smallest validation loss are used for testing. The Adaptive Moment Estimation (ADAM) optimizer was implemented with beta-1 set to 0.9, beta-2 set to 0.999, and an epsilon value to $1\times 10^{-8}$. The learning rate is set to $1\times 10^{-5}$ on both architectures. Data augmentation in real time is used during training, the magnitude of the operations has been set using the optimized values obtained with the Bayesian optimization. The objective function optimized during training is based on the Dice loss and considers the Dice coefficient for the prostate gland segmentation and PZ segmentation as displayed Equation (1).

(1) $$L_{\mathrm{Train}}=1-\frac{2\sum_{i}{\hat{y}}_{ip}y_{ip}}{\sum_{i}{\hat{y}}_{ip}^{2}+\sum_{i}y_{ip}^{2}}+\alpha \left(1-\frac{2\sum_{i}{\hat{y}}_{ipz}y_{ipz}}{\sum_{i}{\hat{y}}_{ipz}^{2}+\sum_{i}y_{ipz}^{2}}\right)$$

where $y_{ip}$ refers to the ground truth value of pixel *i* for the prostate gland segmentation, and ${\hat{y}}_{ip}$ the predicted probability for pixel *i* for the prostate gland segmentation. Similarly, $y_{ipz}$ and ${\hat{y}}_{ipz}$ refers to the ground truth value of pixel *i* and the predicted probability for pixel *i* for PZ segmentation, respectively. Finally, α is a weight parameter that was set to 0.1 after using a random search approach that aims to maximize the whole prostate segmentation Dice. The process to obtain a 2D-3D ensemble PPZ-SegNet is shown in Figure 1, which is composed of 3 steps. In steps 1 and 2, the 2D and 3D CNN were constructed using a Bayesian optimization approach. In step 3 the ensemble is formed by training the 2D and 3D CNN in each of the corresponding folds.

### Experimental design & evaluation criteria

We used part of the largest sample size cohort for training (*n* = 150, Train #1) and the testing data came from 4 different cohorts (*n* = 283, Tests #1–4). Details on the training and testing cohorts are shown in Table 1. Images in the test cohorts are from ProstateX (*n* = 192, not part of training, Test #1), TCIA Prostatectomy (*n* = 26, Test #2), TCIA Repeatability (*n* = 15, Test #3), and Promise12 (*n* = 50, Test #4).

**Table 1:** Description of the patient cohorts used for the study.

	Cohort ID	Data Source	Patient Count	Scan Parameters			
				Manufacturer & Model	Repetition time	Echo time	Field Strength
**Training**							
1	Train#1	Prostate X (Train)	150	Siemens Skyra (*n* = 150)	Median: 5,805 Mean: 6047.6 Stdev: 534.6	Median:104 Mean:103.9 Stdev:0.76	3 T (*n* = 150)
**Testing (data not part of training)**							
1	Test#1	Prostate X (Test)	192	Siemens:-Skyra (*n* = 138) -TrioTim (*n* = 54)	Median: 5,805 Mean: 6047.6 Stdev: 534.6	Median:104 Mean:103.9 Stdev:0.76	3 T (*n* = 192)
2	Test#2	TCIA (Prostatectomy)	26	Phillips Achieva(*n* = 26)	Median Mean:8868.7 Stdev: 0	Median/Mean: 120 Stdv:0	3 T (*n* = 26)
3	Test#3	TCIA (Repeatability)	15	GE Medical System:-Signa HDxt (*n* = 7) -Discovery MR750w (*n* = 8)	Median: 4,546 Mean:4326.2 Std: 577.7	Median: 95.1 Mean:95.97 Std: 6.07	3 T (*n* = 15)
4	Test#4	Promise 12	50	*N/A*	*N/A*	*N/A*	*N/A*

**Figure 2:**
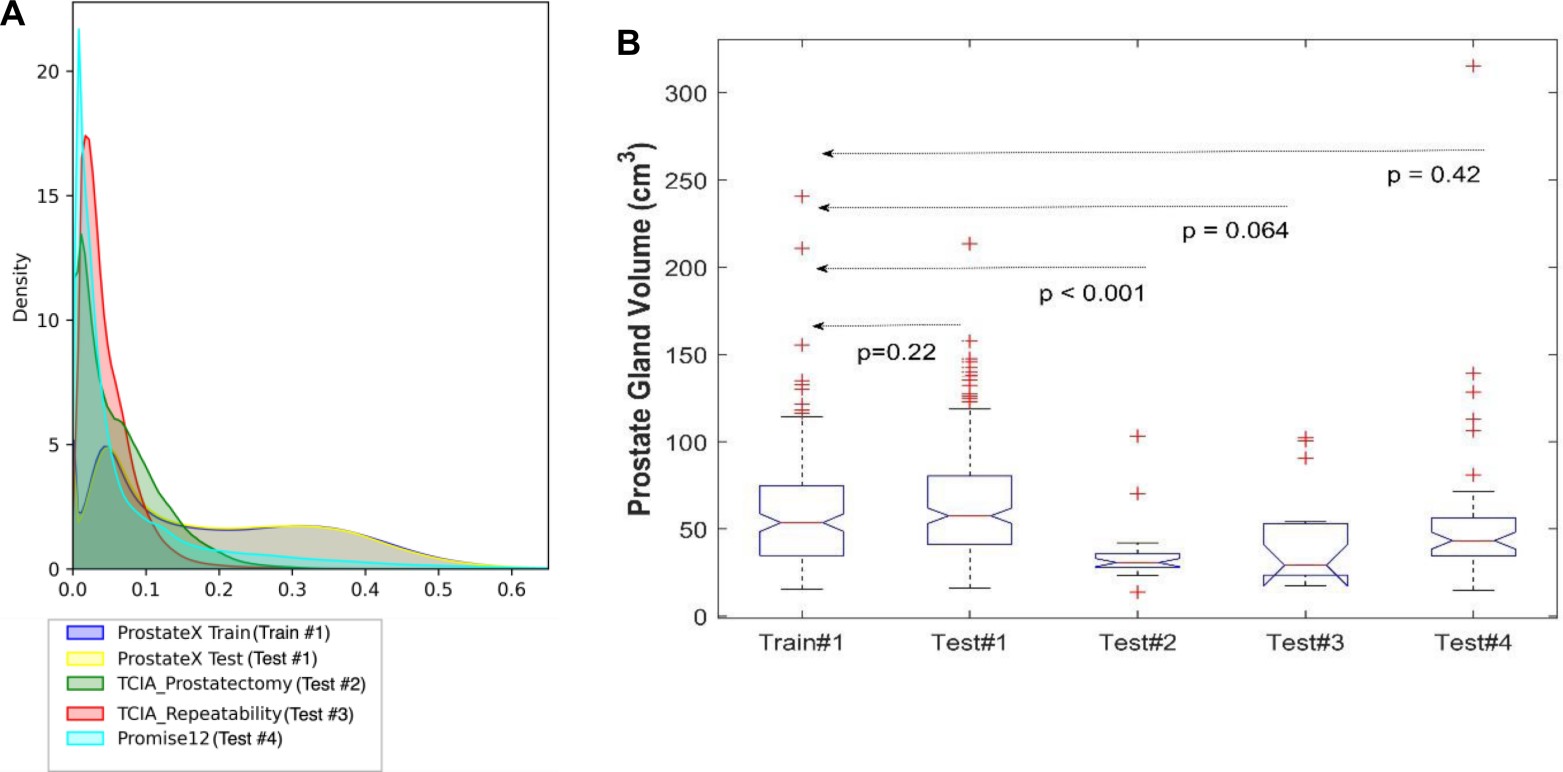
Distribution of the datasets used for model training and testing. (**A**) The pixel intensity distribution of all training and testing sets, (**B**) Box plots of all testing using prostate volume.

To understand the distributions of the datasets, we calculated descriptive statistics on the volumes of gland and PZ for the train and test cohorts as displayed in Tables 2, 3 (also see Supplementary Tables S1, S2). As well, in Figure 2 the pixel intensity distributions of the entire cohort are shown.

**Table 2:** Segmentation performance measured by dice coefficient and corresponding prostate gland volume across the cohorts.

Name index	Gland boundary delineation	Dice Coefficient *(Mean, 95% Confidence, Median)*	Prostate Volume (cm^3^), *(Mean, 95% Confidence, Median)*	Comparison of Volumes *(with Train#1, p-value)*
Train#1	Consensus Radiology read (MCC)	n/a	60.4, [54.85,65.99], 53.6	–
Test#1	Consensus Radiology read (MCC)	0.854, [0.843,0.868], 0.887	64.9, [60.2, 69.6], 57.5	0.22
Test#2		0.794, [0.749,0.839], 0.805	34.9, [28, 41.8], 30.7	<0.001
Test#3		0.806, [0.733,0.88], 0.842	44.2, [ 27.7,60.7], 29.2	0.0635
Test#4	Organizers Provided	0.622, [0.535,0.71], 0.737	54.7, [41.8, 67.6], 43.3	0.418

**Table 3:** Segmentation performance of peripheral zonal (PZ) measured by dice coefficient and corresponding volume across the cohorts.

Name index	PZ boundary	Dice Coefficient (Mean,95% Confidence, Median)	PZ Volume (cm^3^), (Mean, 95% Confidence, Median)
**Train#1**	Consensus Radiology read (MCC)	n/a*	16.2, [15.1,17.3], 14.3
Test#1	Consensus Radiology read (MCC)	0.664, [0.641,0.688], 0.718	18.2, [16.7,19.6], 15.9
Test#2		0.645, [0.563,0.727], 0.686	13.9, [11.2,16.5], 13.5
Test#3		0.640, [0.457,0.717], 0.68	9.24, [ 7.3,11.1], 10.2
Test#4	n/a*	n/a*	n/a*

*n/a, not available. No reference PZ region was available.

Moreover, to analyze the shift between the distributions, 3 discrepancy metrics were calculated among the Train #1 and the 4 testing cohorts. The metrics considered are the Kullback-Leibler divergence, Wasserstein distance, and Jensen Shannon divergence. The obtained values are presented in Table 4.

**Table 4:** Discrepancy metrics between Train #1 and testing cohorts. A higher value means a higher discrepancy between distributions.

Cohort	Discrepancy metric		
	KL-divergence	Wasserstein distance	Jensen Shannon distance
Test #1	0.002	0.001	0.022
Test #2	0.651	0.002	0.420
Test #3	0.892	0.002	0.479
Test #4	0.677	0.002	0.368

To validate the PPZ-SegNet architecture for automatic prostate segmentation in MRI-T2W, 3 observers segmented the prostates manually. The manual segmentations were done slice by slice using MIM Software, and the contours of the prostate were defined without any further algorithmic support. To evaluate the predicted segmentation, we applied widely used indicators in medical imaging for evaluating the segmentation volumes, Dice score (DS) (36) and Hausdorff distance (HD) (37). The DS and HD for each case was calculated using the open-source tool SimpleITK (38, 39). The DS is defined in Equation (2):

(2) $$\mathrm{DS}=\frac{2|y^{1}\cap {\hat{y}}^{1}|}{\left|y^{1}|+|{\hat{y}}^{1}\right|}$$

where |∗| represents the cardinality operator, $y^{1}$ the ground truth voxels from foreground, and ${\hat{y}}^{1}$ the voxels predicted to be part of the foreground. The DS ranges between 0 and 1, where a value of 1 means the network's prediction completely overlaps the ground truth segmentation. Therefore, values closer to 1 mean a better predicted segmentation. Meanwhile, HD is presented in Equation (3).

(3) $$\mathrm{HD}(y,\hat{y})\;=\max(h(y,\hat{y}),\;h(\hat{y},y))$$

where $h(y,\hat{y})$ is the directed HD between the ground truth segmentation *y* and predicted segmentation $\hat{y}$ as defined in Equation (4). HD is a distance measured in mm; a smaller distance means a better segmentation.

(4) $$h(y,\hat{y})\;=\underset{y\;\in \;y^{1}}{\max}\underset{y\;\in \;{\hat{y}}^{1}}{\min}||y-\hat{y}||$$

## Results

We used 4 distinct patient cohorts with over 433 MR scans (150 for training and 283 for testing) in this study. The patient scans were from 3 major manufacturers (i.e., Siemens, Phillips and GE Medical Systems) on 3 T magnet field strength with different resolutions, described in Table 1. Testing was conducted among 4 different cohorts, where no patient samples in the test cohort were part of the training. All testing images underwent the same preprocessing operations described in *Methods* section. The segmentation of gland boundary was provided by consensus reads (Train #1, Test #1, Test #2, and Test #3), and an independent reader (Test #4). The average volume of prostate gland for the training cohort (Train #1) was 60.4 [54.84, 65.99] cm^3^ based on radiologist reference, while the testing cohorts were: (Test #1) 64.9 [60.2, 69.6] cm^3^, (Test #2) 34.9 [28.0, 41.8] cm^3^, (Test #3) 44.2 [27.7, 60.7] cm^3^, (Test # 4) 54.7[41.8, 67.6] cm^3^, please refer to Supplementary Table S1. We found patient glands were diverse in their sizes/volumes across the validation cohorts; a statistical testing (t-test, unpooled) revealed an insignificant difference between Train #1 and Test #1 (*p* = 0.22), Test #4 (*p* = 0.418), borderline with Test #3 (*p* = 0.064), and significant with Test #2 (*p* < 0.001), see Table 2 (and Supplementary Table S1). The average Dice concordance coefficient between the estimated gland volume to the radiologist drawn references for the cohorts were: (Test #1) 0.854 [0.843, 0.868], (Test #2) 0.794 [0.749, 0.839], (Test #3) 0.806 [0.733, 0.88], (Test #4) 0.622 [0.535, 0.71], see Table 2 (and Supplementary Table S1) for details. Figure 2 shows the pixel intensity distribution for the samples across the cohorts. It is evident that Train #1 and Test #1 follow similar intensity distributions, both being bimodal and having a heavy right tail. Meanwhile, the intensity distributions of Tests #2 to #4 are unimodal and most of their density range between pixel intensities 0 and 0.2, different from that for Test #1. Similar results were obtained with the discrepancy metrics, where the Train #1 and Test #1 had the most similar distribution in all metrics. By contrast, the Train #1 and Test #3 cohorts had the greatest discrepancy. Considering the behavior of the Dice coefficient, we can conclude that as the distance (discrepancy) between the training set and the testing set increases, the performance decreases. The results are consistent with previous literature, which shows that even a small change in the testing distribution can make a deep learning model fail during inference (40, 41). Interestingly though, the network's worst performance is in the segmentation of Test #4. In Figure 2A, pixel intensities distribution for Test #4 concentrates on the left but with a light right tail. This means that the contrasts of the images in Test #4 are lower compared to the images in other test cohorts, especially the training cohort.

**Figure 3:**
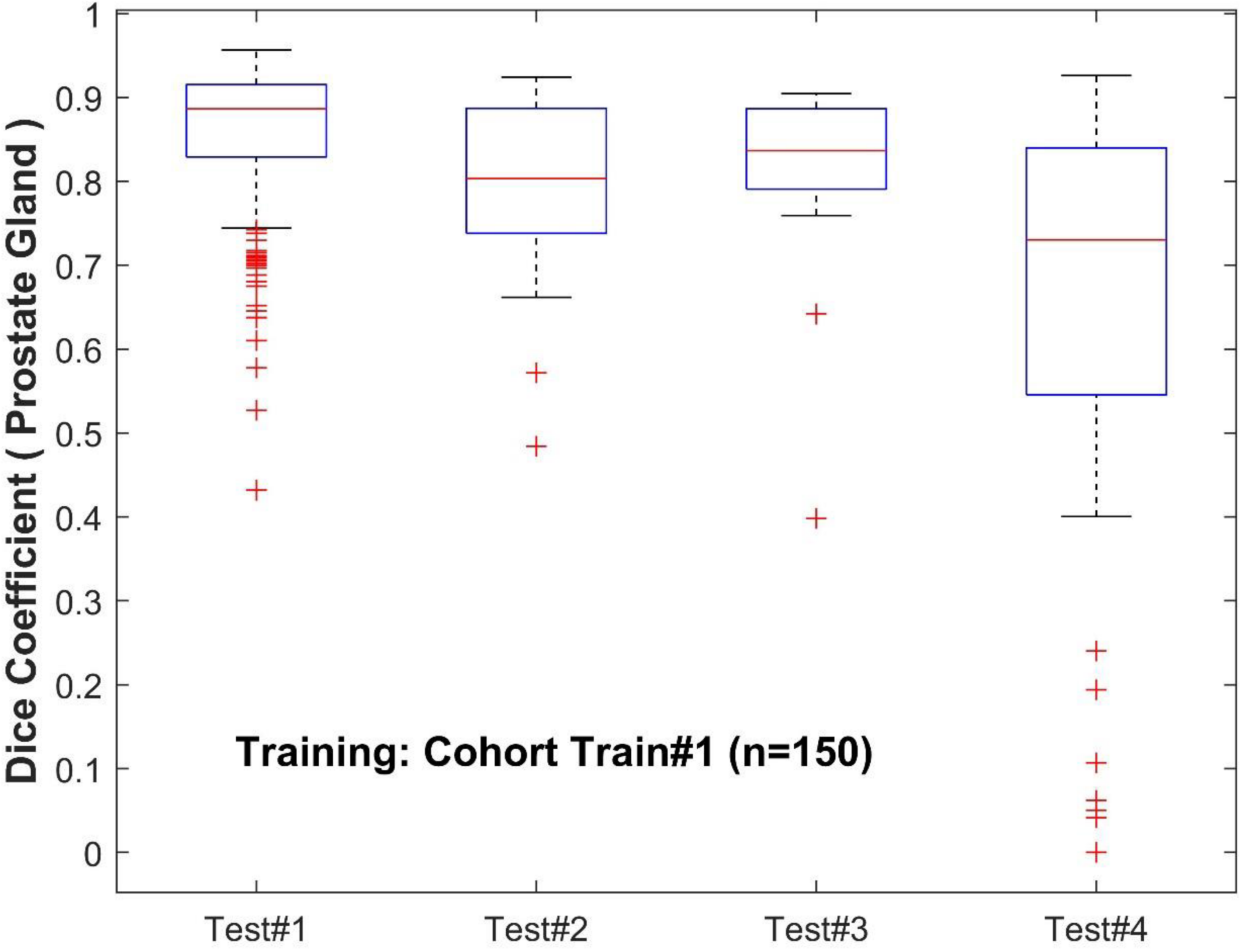
Performance of PPZ-Net for prostate gland segmentation evaluated using dice coefficient across different test cohorts.

**Figure 4:**
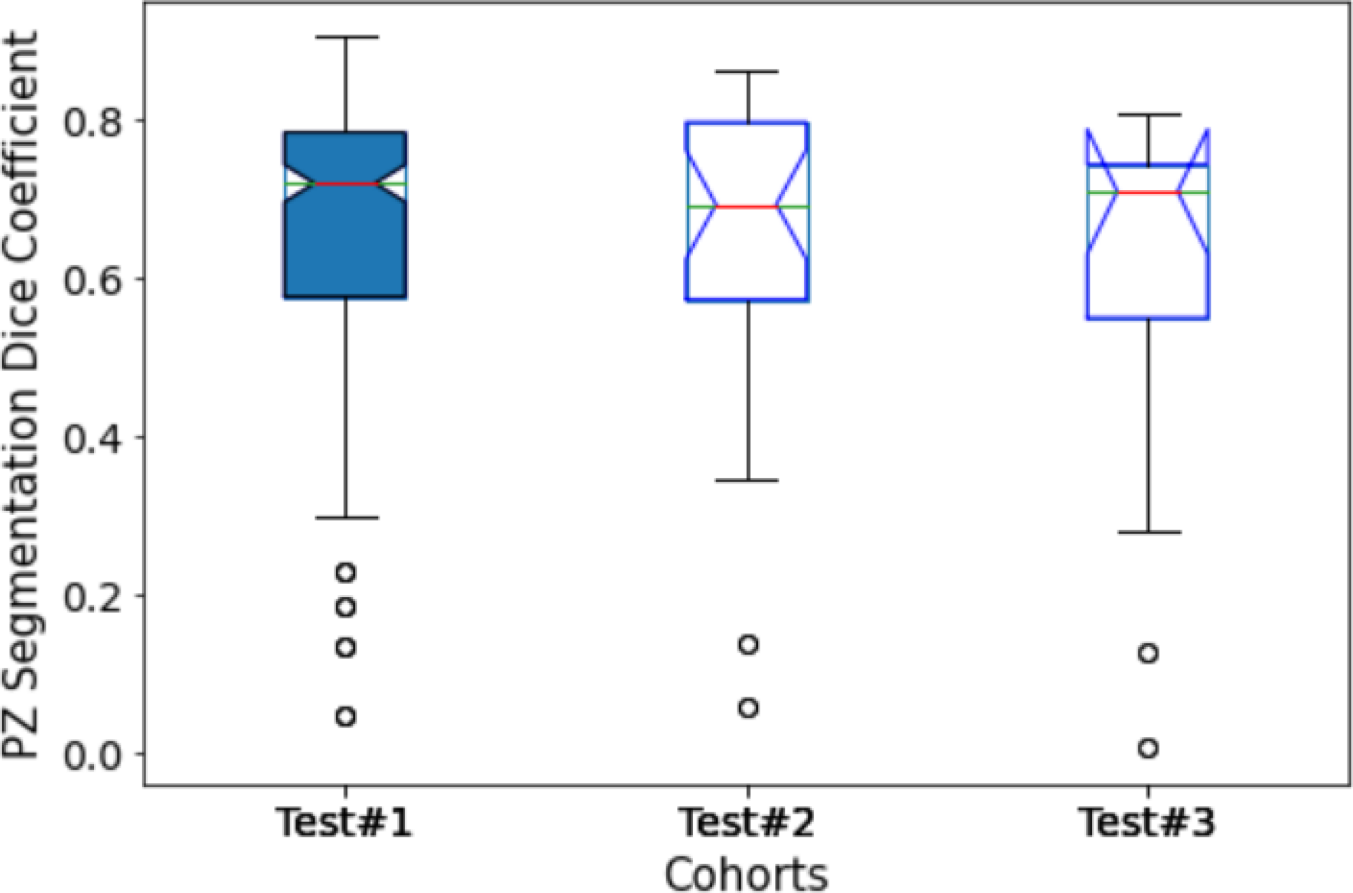
Performance of PPZ-Net for peripheral gland (PZ) segmentation evaluated using dice coefficient across different test cohorts.

To further analyze the performance behavior of the network, the cases in the test cohort were divided into 4 different quartiles based on the patient's gland volume as displayed in Supplementary Figure S1. For each of these quartiles, the corresponding mean, median, and 95% confidence interval of the Dice coefficient were calculated. The results are presented in Supplementary Tables S1, S2 for the prostate gland and PZ segmentation, respectively. The quartiles with the best score for Test #1 are Q2 with a median Dice of 0.895 (mean 0.858) followed by Q4 with a 0.893 of median Dice (mean 0.87). In Test #2, Q4 had the best performance with a 0.916 median Dice (mean 0.883). For Test #3, Q2 had the highest median Dice of 0.875 (mean 0.824). Finally, in Test #4, Q4 achieved the best median Dice of 0.85 (mean 0.77), details are shown in Tables 2, 3 (and Supplementary Tables S1, S2) as well as Figures 3, 4. We evaluated the dependency between the Dice similarity coefficients (between the manual and network provided boundary) and the prostate gland volumes using regression fitted trend lines with confidence bounds. In Supplementary Figure S3, we found that in cohort Test #1 the Dice coefficients are spread across different sizes of the prostate gland measured by its volume. It shows slightly improved trends with higher deviation for larger sized glands. Meanwhile in Tests #2, 3 and 4, there is a marked improvement in the Dice coefficient scores as the gland volumes increase.

Moreover, to analyze the effect of prostate volume in prostate segmentation, the calculated Dice coefficient in each testing cohort is divided into quartiles. The results are presented in Supplementary Figure S4. In Test #1, the boxplots of the Dice coefficients across the quartiles seem to be evenly distributed across the prostate gland volumes, with a small higher incidence of high Dice coefficients in the large prostate gland sizes (volumes). This would mean that the model is able to satisfactorily recognize the prostate gland of prostates with the range of volumes present in Test #1. The reason could be attributed to the similarity in the distributions with the training cohort, and hence the model performance fared better across the population. In Tests #2 and 4, the higher Dice coefficients (*Q*4) are obtained with the highest gland volumes. In Test #3, Dice coefficients in *Q*2 and *Q*4 are obtained from cases with the largest gland volume. This is evident with higher median Dice coefficients seen for the quartile groups with larger glands in most of the test cohorts.

Additionally, we computed the HD between the estimated boundary and the radiologist provided reference. Test #3 had the smallest average HD of 9.47, followed by Test #1 with a mean of 11.33, Test #2 at 12.17, and Test #4 with a mean of 21.73, details are shown in Table 7. Since Test #3 is the dataset with the smallest prostate volumes, it is expected that a distance-based metric such as the Hausdorff will have a lower value than datasets with bigger segmentations. Nevertheless, the results are similar to the ones obtained with the Dice coefficient, where the model had a good performance in Test #1 and the worst performance in Test #4.

**Figure 5:**
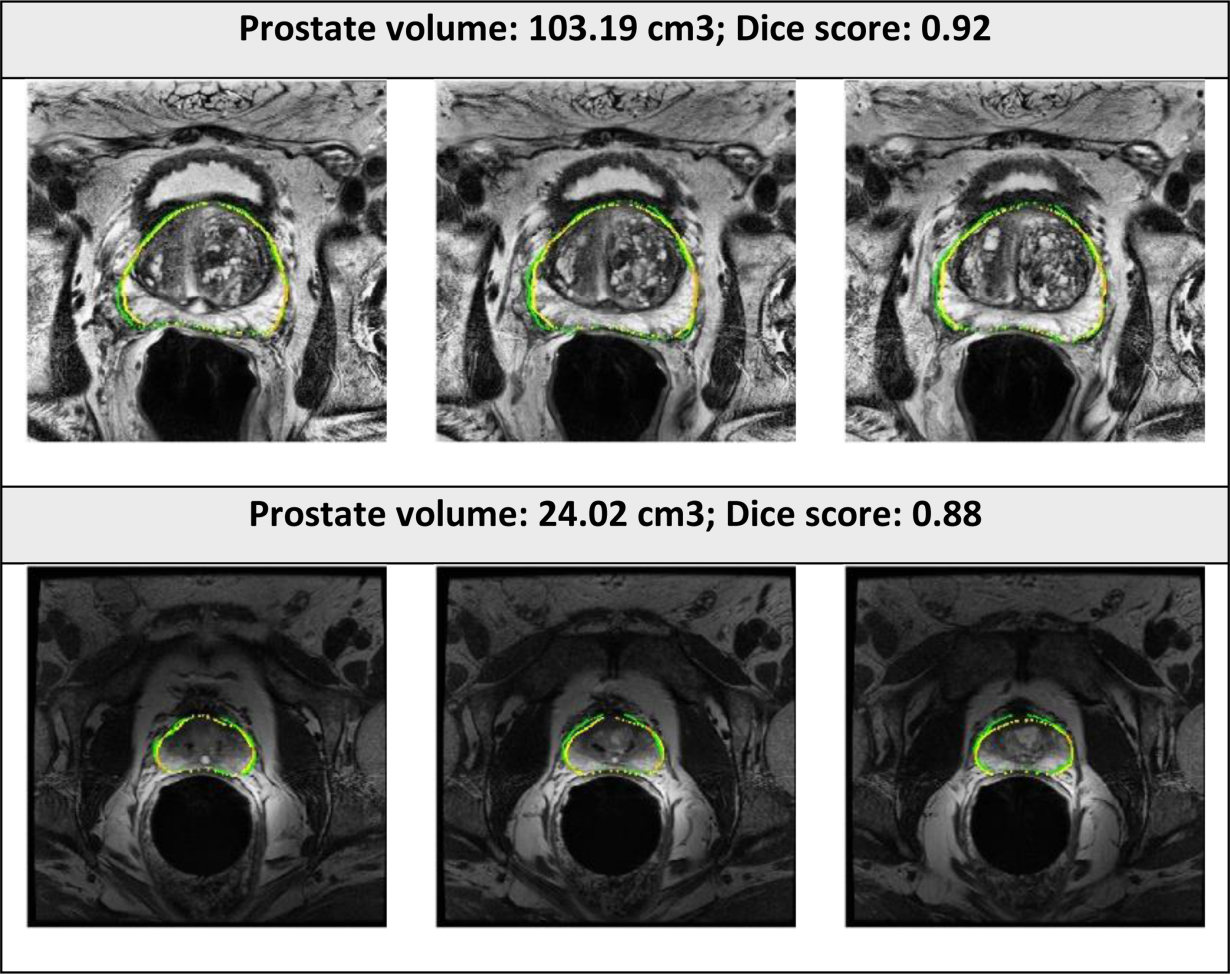
Segmented prostate gland regions with high concordance using PPZ-SegNet (yellow outlines) compared to manual delineation (green) illustrated for selected patient cases with representative axial slices (three consecutive sections).

**Figure 6:**
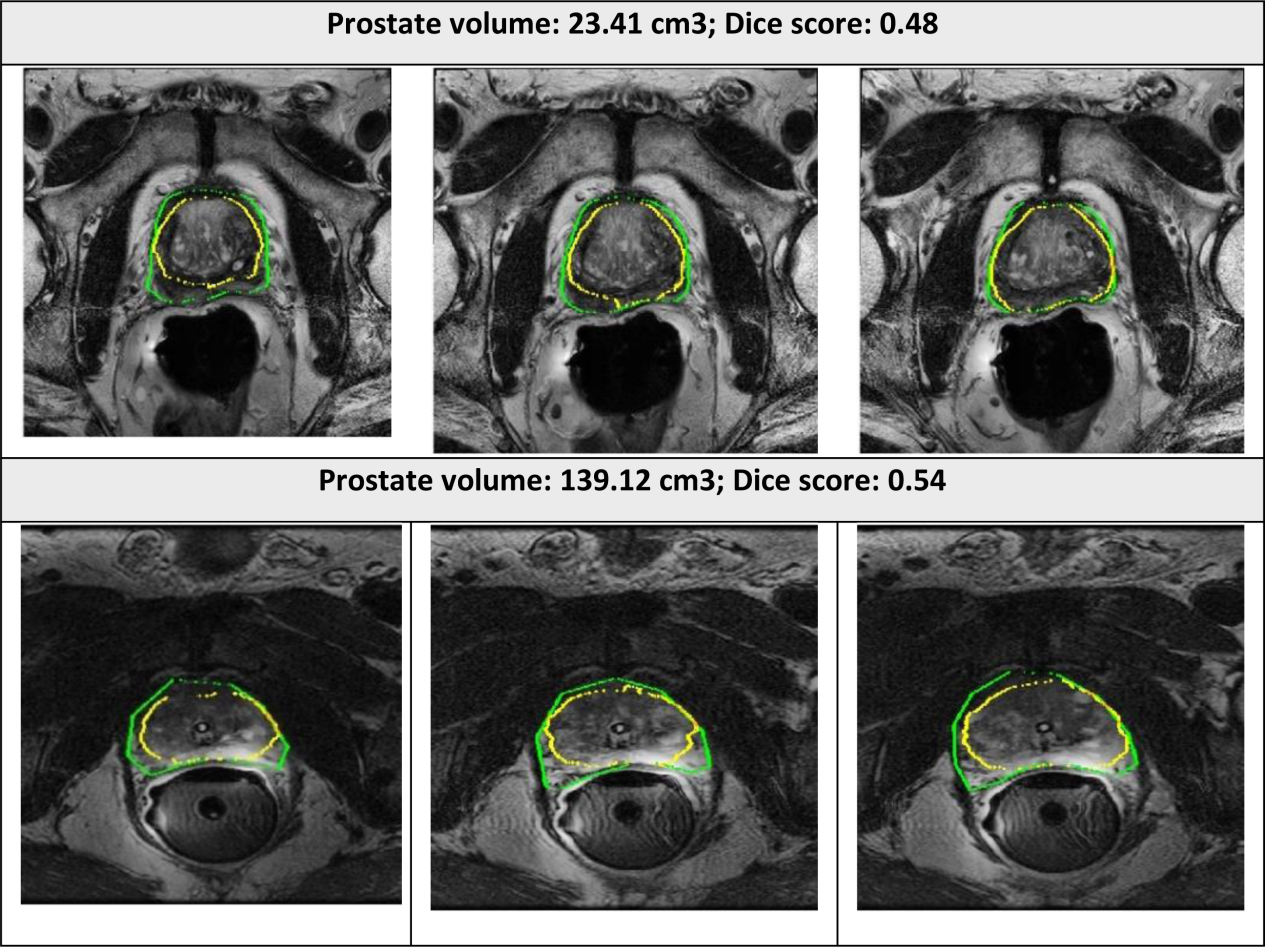
Segmented prostate gland regions with low concordance using PPZ-SegNet (yellow outlines) compared to manual delineation (green) illustrated for selected patient cases with representative axial slices (three consecutive sections).

The examples of good and bad segmentation results of the network are displayed in Figures 5, 6. The network correctly delineates regular- and irregular-shaped prostates. However, sometimes it fails to provide a continuous contour and identify the prostate region in images with low contrast.

### Dataset composition analysis

An important factor in domain adaptation is the composition of the training dataset. Therefore, we tested how the performance of the PPZ-SegNet changed when we modified the training set. First, the PPZ-SegNet was trained on 30 cases (Test #2: *n* = 20; Test #3: *n* = 10) and tested in 253 cases (Test #1: *n* = 192; Test #2: *n* = 6; Test #3: *n* = 5; Test #4: *n* = 50). None of the testing cases were used for training. We denote this model as PPZ-SegNet_TCIA. The evaluation metrics are presented in Table 5A. In comparison to the results obtained with the PPZ-SegNet trained only with Train #1, which we call PPZ-SegNet_PX, the Dice coefficients reduced for all testing datasets with exception of Test #3. Since Train #1 has a larger number of images and a more varied size of prostate glands, it provides the network with more information to generalize better to the other testing datasets. An interesting finding is that the PPZ-SegNet_PX performs better than PPZ-SegNet_TCIA in Test #2, even though the latter model is trained using images from Test #2. This might be caused by the reduced number of cases in Test #2 that might not completely characterizes the distribution of prostates present in that dataset. Moreover, it shows that for Test#2 the PPZ-SegNet_PX achieved domain adaptation.

**Table 5:** Segmentation performance of PPZ-SegNet model measured by Dice coefficients in the following scenario. (**A**) Smaller cohort training (*n* = 30 cases, using part of Test#2, *n* = 20 and Test#3, *n* = 10), tested on remaining 253 cases. (**B**) Larger cohort training (*n* = 91, using all of Train#1, Test#2, and Test#3), tested on remaining 242 cases (Test #1 & Test #4).

Cohort	Prostate gland segmentation	PZ segmentation
**(A) Smaller cohort training (*n* = 30 cases)**		
Test #1	0.699	0.409
Test #2	0.758	0.608
Test #3	0.876	0.655
Test #4	0.567	n/a
**(B) Larger cohort training (*n* = 91 cases)**		
Test #1	0.845	0.647
Test #4	0.695	n/a

For the second experiment we performed, the PPZ-SegNet was trained on 91 cases (Train #1: subsampled *n* = 50; Test #2: *n* = 26; Test #3: *n* = 15) and evaluated on 242 testing cases (Test #1: *n* = 192; Test #4: *n* = 50). We denote this model as PPZ-SegNet_TCIAPX. The evaluation metrics for PPZ-SegNet_TCIAPX are presented in Table 5B. PPZ-SegNet_TCIAPX has the leading performance in Test #4, increasing 7.3% in the Dice coefficient from PPZ-SegNet_PX. Since Test #4 has a high number of cases with small volume sizes, enriching Train #1 with datasets that share the same characteristic (such as Tests #2 and 3) improves the segmentation accuracy. Nevertheless, the Dice coefficient in Test #4 is still low (average 0.695), which shows that there are other imaging characteristics in that specific dataset that affect the performance of network. Moreover, it also demonstrates that domain shift is a difficult problem to solve. In Test #1, PPZ_SegNet_TCIAPX had a slightly lower Dice coefficient than PPZ_SegNet_PX. The reduction is of 1% and 0.7% on the segmentation of prostate gland and PZ area, respectively. This is an expected outcome given that we reduced the number of training cases from Train #1.

### Ensemble analysis

Previous work has shown that ensemble networks obtain better generalization performance than individual networks (42, 43) and other generalization techniques (44). In this section, we analyze the effect ensemble learning has over domain shift. First, we evaluate the effect of having a 2D-3D ensemble (PPZ-SegNet) over a unique 2D ensemble or 3D ensemble. For this objective, we produce the segmentations with the 2D ensemble and 3D ensemble and compare them against the reference segmentations. The evaluation metrics are presented in Table 6. Furthermore, a one-tailed paired t-test with a 95% confidence level is applied to compare the mean performance, the approach has been previously presented (32). The model with the best performance is shown in boldface. The experimental results show that the 2D ensemble performs better than the 3D ensemble in most testing datasets when considering the Dice coefficient. Only in the segmentation of the PZ structure from Test #2, the 3D ensemble has a statistically higher DS. PPZ-SegNet, by contrast, has an equal or better performance than the 2D ensemble, with exception of the prostate gland segmentation in Test #4.

**Table 6:** Evaluation metrics for the predicted prostate gland and PZ segmentation using the 2D ensemble, 3D ensemble, and 2D–3D ensemble (PPZ-SegNet). Best performing model selected using a one-tailed pair-t test [see prior publication, Balderon (32)], selected models Dice performance index highlighted in bold.

Cohort	Dice Coefficient					
	Prostate gland segmentation			PZ segmentation		
	2D Ensemble	3D Ensemble	PPZ-SegNet	2D Ensemble	3D Ensemble	PPZ-SegNet
Test #1	0.850	0.851	**0** **.** **855**	0.654	0.655	**0** **.** **664**
Test #2	**0** **.** **795**	0.783	**0** **.** **794**	0.624	**0** **.** **638**	**0** **.** **645**
Test #3	**0** **.** **793**	0.769	**0** **.** **806**	0.611	0.554	**0** **.** **640**
Test #4	**0** **.** **668**	0.351	0.621	n/a	n/a	n/a
Cohort	**Hausdorff Distance**					
	**Prostate gland segmentation**			**PZ segmentation**		
	2D Ensemble	3D Ensemble	PPZ-SegNet	2D Ensemble	3D Ensemble	PPZ-SegNet
Test #1	**11** **.** **392**	11.583	**11** **.** **325**	16.791	**16** **.** **335**	**16** **.** **314**
Test #2	**12** **.** **003**	13.613	**12** **.** **167**	14.437	**14** **.** **220**	**13** **.** **458**
Test #3	**10** **.** **060**	16.230	**9** **.** **468**	**12** **.** **352**	17.868	**11** **.** **683**
Test #4	**20** **.** **771**	40.569	**21** **.** **729**	n/a	n/a	n/a

Considering the HD, we found the 2D ensemble better than the 3D ensemble in the segmentation of the prostate gland. Meanwhile, the 3D ensemble performs better than the 2D ensemble in the segmentation of the PZ. PPZ-SegNet takes advantage of the 2D and 3D information and is always equal to the best performing ensemble. These results demonstrate that forming a 2D-3D ensemble does slightly improve the generalization capability of a network to different testing cohorts.

We also analyzed how the number of networks in the ensemble affect domain adaptation. The results are presented in Table 7, where we vary the number of 2D–3D ensembles from 1 to 5 and compare the resulting segmentations to the reference segmentation. A one-tailed paired t-test with a 95% confidence is also applied to statistically compare the results. In terms of the Dice coefficient, the number of ensemble networks does not seem to affect the network's performance as in most testing datasets their performance is statistically the same. Bearing in mind the HD, the PPZ-SegNet has a statistically smaller HD in the segmentation of the prostate gland than ensembles with a lower number of networks. Therefore, we conclude that increasing the number of networks in the ensemble only improves the generalization capability when segmenting the prostate gland and using the HD as an evaluation metric.

**Table 7:** Evaluation metrics for the predicted prostate gland and PZ segmentation using one to five 2D–3D ensembles (denoted as En.). Best performing model selected using a one-tailed pair-*t* test [see prior publication, Balderon (32)], selected models Dice performance index highlighted in bold.

Cohort	Dice Coefficient									
	Prostate gland segmentation					PZ segmentation				
	1 En.	2 En.	3 En.	4 En.	PPZ-SegNet	1 En.	2 En.	3 En.	4 En.	PPZ-SegNet
Test #1	**0** **.** **854**	**0** **.** **855**	**0** **.** **858**	**0** **.** **859**	**0** **.** **855**	**0** **.** **662**	**0** **.** **657**	**0** **.** **667**	**0** **.** **667**	**0** **.** **664**
Test #2	0.778	0.777	0.786	0.783	**0** **.** **794**	**0** **.** **640**	**0** **.** **633**	**0** **.** **645**	**0** **.** **644**	**0** **.** **645**
Test #3	**0** **.** **788**	**0** **.** **784**	**0** **.** **778**	**0** **.** **780**	**0** **.** **806**	**0** **.** **625**	0.560	**0** **.** **638**	**0** **.** **638**	**0** **.** **640**
Test #4	0.625	0.588	**0** **.** **643**	**0** **.** **643**	0.621	n/a	n/a	n/a	n/a	n/a
Cohort	**Hausdorff Distance [mm]**									
	**Prostate gland segmentation**					**PZ segmentation**				
	1 En.	2 En.	3 En.	4 En.	PPZ-SegNet	1 En.	2 En.	3 En.	4 En.	PPZ-SegNet
Test #1	**11** **.** **638**	11.735	**11** **.** **443**	**11** **.** **536**	**11** **.** **325**	**16** **.** **673**	**16** **.** **730**	**16** **.** **272**	**16** **.** **206**	**16** **.** **314**
Test #2	14.997	15.045	13.964	14.112	**12** **.** **167**	14.586	14.518	**14** **.** **152**	**14** **.** **471**	**13** **.** **458**
Test #3	19.441	19.289	16.790	17.242	**9** **.** **468**	14.945	19.959	**11** **.** **474**	12.338	**11** **.** **683**
Test #4	34.350	36.798	28.669	30.379	**21** **.** **729**	n/a	n/a	n/a	n/a	n/a

## Discussion

The present study implements a modified deep neural network architecture, based on the architecture previously published (32), refer to as the PPZ-SegNet. This network was optimized and trained using a cohort of 150 patients (Train #1) with T2W-MR 3D imaging data and tested in 4 different cohorts with diverse gland sizes, distributed across the cohorts: Test #1 (*n* = 192), Test #2 (*n* = 26), Test #3 (*n* = 15), Test #4 (*n* = 50). This study uniquely evaluates the performance of a deep network on a large independent cohort that was not part of training. The proposed method provides both the gland-segmentation region and the PZ. It is well documented that most tumors located in the prostate appear in the PZ region of the glands, estimated to be over 70% (45), hence the assessment of the zonal boundary determination makes it necessary.

Our study uses T2W MR images to segment the regions in 3D volumes, the native resolution is mapped to a uniform resolution before being used as an input to the network. In the preprocessing step, input images are standardized to a fixed resolution of 0.5 × 0.5 × 3 mm, which is a necessary step followed in most network architectures to reduce detection biases. In our study, we use a dual network architecture that uses both the 2D and 3D volumes of the MR T2W images to generate an independent assessment and obtain a consensus to converge on a boundary that captures the glandular structure (see Figure 1). Prior studies show better segmentation performance by using a combined architecture than training with a single data stream (32, 46), results that have been confirmed in our experiments. Our study network provides a best average Dice coefficient of 0.855 [0.843, 0.868] for Test #1 (ProstateX). The reproducible results across 4 diverse cohorts, are with average Dice of 0.794 [0.749, 0.839] in Test #2, 0.806 [0.733, 0.88] in Test #3 and 0.622 [0.55, 0.71] in Test #4, respectively.

A recent published deep model (47) (ProGNet) reports optimistic results with a claim that the model is reproducible in an independent cohort. The model was trained on 805 prostate mpMR-T2W images and reported a Dice coefficient in the range of 0.92 (*n* = 100, internal cohort), 0.87 (*n* = 26, external cohort), 0.89 (*n* = 30, external Promise 12 cohort) to 0.93 (*n* = 11, cohort). The model used a 2.5D network architecture with representative slices (3 slices in a patient) for model training/testing. It becomes challenging to assess the model performance in a very small test-cohort (test cohort size in the range of 12.4% to 1.3% of training size).

In our analysis we show that as the prostate gland volume increases the network seems to perform a better delineation, with a Dice coefficient improving in value. It can be attributed to these factors. First, the training data had patients with larger glandular volumes making the network perform better at a larger size; and second, the test cohort patients' glandular volumes span smaller size ranges that the network has not seen during the training phase. The figures and the tables show the relationship of the network models between the glandular volume and its delineation performance metrics (see Supplementary Figures S1, S2; Supplementary Tables S1, S2). Differently from other works, we show the performance of our network in other testing cohorts. This should be a common practice as it demonstrates the robustness and limitations of the proposed networks.

An important analysis performed in this work is the effect that the composition of the training set has in domain adaptation. The PPZ-SegNet model trained with 150 cases from Train #1 had a better generalization capability than the PPZ-SegNet model trained with 30 cases from Test #2 and Test #3 (refer to Table 5A). This demonstrates that having a larger and more diverse dataset improves the performance on new cohorts. Furthermore, enriching Test #2 and Test #3 with 50 cases from Train #1 produced the best DS in Test #4 as shown in Table 5B. This can be attributed to the wider range of prostate volumes considered during training.

In this work we also demonstrate that ensemble of 2D and 3D network can be a helpful technique to improve the generalization to new cohorts. Our experiments displayed in Table 6, show that using a 2D-3D ensemble provides a slight better segmentation performance than a unique 2D or 3D ensemble on all testing cohorts. 2D and 3D networks extract distinct levels of information, and each one will have a better performance on certain types of datasets and segmentation tasks. In our experiments, the 2D ensemble leads in the segmentation of the prostate gland in terms of the Dice coefficient and HD. Similar results have already been obtained in other works (26), where 3D networks have a lower performance than 2D networks on datasets with a high inter-slice resolution as the distance makes the information of nearby slices less relevant to predict the current segmentation. Meanwhile, the 3D network performs better in the segmentation of the PZ when considering the Haussdorff evaluation metric. The PPZ-SegNet takes advantage of both types of architectures and obtains the same results as the best performing network in all testing sets and evaluation metrics. As it is unknown what will be the characteristics of future testing cohorts, it is a good tactic to use a 2D–3D ensemble that will be able to exploit different types of feature relationships.

Another result from our experiments is that increasing the number of 2D–3D ensembles from 1 to 5 improves generalization when segmenting the prostate gland and using the HD as evaluation metric. Given that training five 2D–3D ensembles over one is computationally costly, it is necessary to analyze if the improvement justifies the additional cost. In our experiments, using the PPZ-SegNet over the one 2D–3D ensemble reduced the Haussdorff distance from 19.44 mm to 9.46 mm in Test #3 and from 34.35 mm to 21.73 mm in Test #4, which is a significant improvement. Hence for these cohorts using the PPZ-SegNet is justified.

Variability among expert readers in manual annotation of the prostate/zonal anatomy and poor image quality are possible factors for poor performance of deep neural networks, which could cause domain shift, a common problem in machine learning, resulting in inappropriate models across cohorts (27, 41, 48). This could impact segmentation performance, possible reasons for lower Dice score in some of the test cohorts (Test #4). On the other hand, we also noticed that Test #1 achieved the highest average Dice score among all test cohorts, and this cohort follows a similar intensity distribution to Train #1. This implies that the “standardization” of the training and testing cohorts could lead to more consistent automated segmentations results, suggesting that the MRI-T2W of prostate should follow the PI-RADS guideline. In addition, some image pre-processing procedures, such as histogram matching, may also help. We will investigate this further.

A limitation in our study is that we compared the PPZ-SegNet segmentation results with the manual annotation by our research radiologists using metrics like the DS. Although we tried to minimize the manual contouring variabilities through the consensus reading between the research and clinical radiologists, the intra- and inter-operator variabilities in manual annotations do exist (49) and would require further evaluation to assess its influence between readers, across institutions. Another limitation when constructing the PPZ-SegNet is the need to apply partial training when implementing the Bayesian Optimization. Although we tested the number of training epochs that provided a good approximation of the final performance, this strategy might bias the selection of fast learning architectures instead of best performing networks.

## Conclusion

We proposed an automatic DL method (PPZ-SegNet) for segmentation of the prostate and its PZ on MRI-T2W. The proposed models use the Bayesian Optimization approach to minimize the segmentation errors and less trainable parameters compared to conventional U-net architecture. Our study finds performance of the neural networks trained under limited training data could degrade when they are applied on the images that are different from the training data, we show there is a dependency with respect to prostate gland sizes. It is possible there are other parameters that our study does not have the statistical power to evaluate. Furthermore, we found that using a 2D-3D ensemble slightly improves the generalization capability of a network. Future work is needed to investigate the capabilities of such networks on datasets with different types of variations and explore other networks which is more invariant to differences in the input data.

## Data Availability

The datasets used in this study and the model presented can be found at the following online repositories: The Cancer Imaging Archive (TCIA): https://www.cancerimagingarchive.net/; Promise 12 Challenge: https://promise12.grand-challenge.org/; Prostate X Challenge: https://wiki.cancerimagingarchive.net/pages/viewpage.action?pageId=23691656; and the Deep leaning model code: https://github.com/mariabaldeon/PPZ-SegNet.git.

## References

[1] Rawla P. Epidemiology of prostate cancer. World J Oncol. (2019) 10(2):63–89. 10.14740/wjon1191 31068988 PMC6497009

[2] Sung H Ferlay J Siegel RL Laversanne M Soerjomataram I Jemal A Global cancer statistics 2020: GLOBOCAN estimates of incidence and mortality worldwide for 36 cancers in 185 countries. CA Cancer J Clin. (2021) 71(3):209–49. 10.3322/caac.21660 33538338

[3] Mendhiratta N Taneja SS Rosenkrantz AB. The role of MRI in prostate cancer diagnosis and management. Future Oncol. (2016) 12(21):2431–43. 10.2217/fon-2016-0169 27641839

[4] Johnson LM Turkbey B Figg WD Choyke PL. Multiparametric MRI in prostate cancer management. Nat Rev Clin Oncol. (2014) 11(6):346–53. 10.1038/nrclinonc.2014.69 24840072 PMC6330110

[5] Grivas N Hinnen K de Jong J Heemsbergen W Moonen L Witteveen T Seminal vesicle invasion on multi-parametric magnetic resonance imaging: correlation with histopathology. Eur J Radiol. (2018) 98:107–12. 10.1016/j.ejrad.2017.11.013 29279147

[6] Zaorsky NG Showalter TN Ezzell GA Nguyen PL Assimos DG D'Amico AV ACR Appropriateness criteria for external beam radiation therapy treatment planning for clinically localized prostate cancer, part II of II. Adv Radiat Oncol. (2017) 2(3):437–54. 10.1016/j.adro.2017.03.003 29114613 PMC5605284

[7] Pathmanathan AU van As NJ Kerkmeijer LGW Christodouleas J Lawton CAF Vesprini D Magnetic resonance imaging-guided adaptive radiation therapy: a “game changer” for prostate treatment? Int J Radiat Oncol Biol Phys. (2018) 100(2):361–73. 10.1016/j.ijrobp.2017.10.020 29353654

[8] Marks L Young S Natarajan S. MRI-ultrasound fusion for guidance of targeted prostate biopsy. Curr Opin Urol. 2013;23(1):43–50. 10.1097/MOU.0b013e32835ad3ee 23138468 PMC3581822

[9] Volkin D Turkbey B Hoang AN Rais-Bahrami S Yerram N Walton-Diaz A Multiparametric magnetic resonance imaging (MRI) and subsequent MRI/ultrasonography fusion-guided biopsy increase the detection of anteriorly located prostate cancers. BJU Int. (2014) 114(6b):E43–E9. 10.1111/bju.12670 24712649 PMC5613950

[10] Perera M Krishnananthan N Lindner U Lawrentschuk N. An update on focal therapy for prostate cancer. Nat Rev Urol. (2016) 13(11):641–53. 10.1038/nrurol.2016.177 27670618

[11] Smith WL Lewis C Bauman G Rodrigues G D'Souza D Ash R Prostate volume contouring: a 3D analysis of segmentation using 3DTRUS, CT, and MR. Int J Radiat Oncol. (2007) 67(4):1238–47. 10.1016/j.ijrobp.2006.11.027 17336224

[12] Klein S van der Heide UA Lips IM van Vulpen M Staring M Pluim JP. Automatic segmentation of the prostate in 3D MR images by atlas matching using localized mutual information. Med Phys. (2008) 35(4):1407–17. 10.1118/1.2842076 18491536

[13] Toth R Madabhushi A. Multifeature landmark-free active appearance models: application to prostate MRI segmentation. IEEE Trans Med Imaging. (2012) 31(8):1638–50. 10.1109/TMI.2012.2201498 22665505

[14] Zheng Y Comaniciu D. Marginal space learning for medical image analysis. Berlin: Springer (2014). 10.1016/j.media.2012.09.007 23265800

[15] Makni N Iancu A Colot O Puech P Mordon S Betrouni N. Zonal segmentation of prostate using multispectral magnetic resonance images. Med Phys. (2011) 38(11):6093–105. 10.1118/1.3651610 22047374

[16] Litjens G Debats O van de Ven W Karssemeijer N Huisman H. A pattern recognition approach to zonal segmentation of the prostate on MRI. Med Image Comput Comput Assist Interv. (2012) 15(Pt 2):413–20. 10.1007/978-3-642-33418-4_51 23286075

[17] Tran KA Kondrashova O Bradley A Williams ED Pearson JV Waddell N. Deep learning in cancer diagnosis, prognosis and treatment selection. Genome Med. (2021) 13(1):152. 10.1186/s13073-021-00968-x 34579788 PMC8477474

[18] Litjens G Kooi T Bejnordi BE Setio AAA Ciompi F Ghafoorian M A survey on deep learning in medical image analysis. Med Image Anal. (2017) 42:60–88. 10.1016/j.media.2017.07.005 28778026

[19] Hesamian MH Jia W He X Kennedy P. Deep learning techniques for medical image segmentation: achievements and challenges. J Digit Imaging. (2019) 32(4):582–96. 10.1007/s10278-019-00227-x 31144149 PMC6646484

[20] Poggio T Banburski A Liao Q. Theoretical issues in deep networks. Proc Natl Acad Sci U S A. (2020) 117(48):30039–45. 10.1073/pnas.1907369117 32518109 PMC7720221

[21] Gao J Jiang Q Zhou B Chen D. Convolutional neural networks for computer-aided detection or diagnosis in medical image analysis: an overview. Math Biosci Eng. (2019) 16(6):6536–61. 10.3934/mbe.2019326 31698575

[22] Krizhevshy A Sutskever I Hilton GE. ImageNet classification with deep convolutional neural networks. Commun ACM. (2017) 60(6):84–90. 10.1145/3065386

[23] LeCun Y Bengio Y Hinton G. Deep learning. Nature. (2015) 521(7553):436–44. 10.1038/nature14539 26017442

[24] Rajpurkar P Irvin J Ball RL Zhu K Yang B Mehta H Deep learning for chest radiograph diagnosis: a retrospective comparison of the CheXNeXt algorithm to practicing radiologists. PLoS Med. (2018) 15(11):e1002686. 10.1371/journal.pmed.1002686. PubMed PMID: 30457988; PMCID: PMC6245676 following competing interests: CPL holds shares in whiterabbit.ai and Nines.ai, is on the Advisory Board of Nuance Communications and on the Board of Directors for the Radiological Society of North America, and has other research support from Philips, GE Healthcare, and Philips Healthcare. MPL holds shares in and serves on the Advisory Board for Nines.ai. None of these organizations have a financial interest in the results of this study. 30457988 PMC6245676

[25] Isensee F Jaeger PF Kohl SAA Petersen J Maier-Hein KH. nnU-Net: a self-configuring method for deep learning-based biomedical image segmentation. Nat Methods. (2021) 18(2):203–11. 10.1038/s41592-020-01008-z 33288961

[26] . edited by Isensee F Jaeger PF Full PM Wolf I Engelhardt S Maier-Hein KH. Automatic cardiac disease assessment on cine-MRI via time-series segmentation and domain specific features. Cham: Springer International Publishing (2018).

[27] Ben-David S Blitzer J Crammer K Kulesza A Pereira F Vaughan JW. A theory of learning from different domains. Mach Learn. (2010) 79(1):151–75. 10.1007/s10994-009-5152-4

[28] Halfpenny W Baxter SL. Towards effective data sharing in ophthalmology: data standardization and data privacy. Curr Opin Ophthalmol. (2022) 33(5):418–24. 10.1097/icu.0000000000000878 35819893 PMC9357189

[29] Balachandar N Chang K Kalpathy-Cramer J Rubin DL. Accounting for data variability in multi-institutional distributed deep learning for medical imaging. J Am Med Inform Assoc. (2020) 27(5):700–8. 10.1093/jamia/ocaa017 32196092 PMC7309257

[30] Alzubaidi L Al-Amidie M Al-Asadi A Humaidi AJ Al-Shamma O Fadhel MA Novel transfer learning approach for medical imaging with limited labeled data. Cancers (Basel). (2021) 13(7):1590. 10.3390/cancers13071590 33808207 PMC8036379

[31] Yu X Wang J Hong Q-Q Teku R Wang S-H Zhang Y-D. Transfer learning for medical images analyses: a survey. Neurocomputing. (2022) 489:230–54. 10.1016/j.neucom.2021.08.159

[32] Baldeon Calisto M Lai-Yuen SK. AdaEn-Net: an ensemble of adaptive 2D-3D fully convolutional networks for medical image segmentation. Neural Netw. (2020) 126:76–94. 10.1016/j.neunet.2020.03.007 32203876

[33] Ren P Xiao Y Chang X Huang PY Li Z Chen X A comprehensive survey of neural architecture search: challenges and solutions. ACM Comput Surv. (2021) 54(4):1–34. 10.1145/3447582

[34] Liu Y Sun Y Xue B Zhang M Yen GG Tan KC. A survey on evolutionary neural architecture search. IEEE Trans Neural Netw Learn Syst. (2021):1–21. 10.1109/TNNLS.2021.3100554 34357870

[35] Mlynarski P Delingette H Criminisi A Ayache N. 3D Convolutional neural networks for tumor segmentation using long-range 2D context. Comput Med Imaging Graph. (2019) 73:60–72. 10.1016/j.compmedimag.2019.02.001 30889541

[36] Taha AA Hanbury A. Metrics for evaluating 3D medical image segmentation: analysis, selection, and tool. BMC Med Imaging. (2015) 15(1):29. 10.1186/s12880-015-0068-x 26263899 PMC4533825

[37] Crum WR Camara O Hill DL. Generalized overlap measures for evaluation and validation in medical image analysis. IEEE Trans Med Imaging. (2006) 25(11):1451–61. 10.1109/tmi.2006.880587 17117774

[38] Tools#1 I-. ITK - Labeloverlapmeasure (2022). Available at: https://simpleitk.org/doxygen/latest/html/classitk_1_1simple_1_1LabelOverlapMeasuresImageFilter.html.

[39] Tools#2 I. ITK Tools HausdorffDistance2022.

[40] Guan H Liu M. Domain adaptation for medical image analysis: a survey. IEEE Trans Biomed Eng. (2022) 69(3):1173–85. 10.1109/tbme.2021.3117407 34606445 PMC9011180

[41] Pooch E, Ballester P, Barros R. Can we trust deep learning based diagnosis? The impact of domain shift in chest radiograph classification. *MICCAI 2020 Proceedings*. Lima, Peru: Springer International Publishing (2020). p. 74–83.

[42] Ganaie MA Hu M Malik AK Tanveer M Suganthan PN. Ensemble deep learning: a review. Eng Appl Artif Intell. 2022;115:105151. 10.1016/j.engappai.2022.105151

[43] Zhang S Liu Meng Yan J. The diversified ensemble neural network. In NIPS’20: proceedings of the 34th international conference on neural information Processing2020. p. 16001–11.

[44] Gencay R Min Q. Pricing and hedging derivative securities with neural networks: bayesian regularization, early stopping, and bagging. IEEE Trans Neural Netw. (2001) 12(4):726–34. 10.1109/72.935086 18249908

[45] Fine SW Reuter VE. Anatomy of the prostate revisited: implications for prostate biopsy and zonal origins of prostate cancer. Histopathology. (2012) 60(1):142–52. 10.1111/j.1365-2559.2011.04004.x 22212083

[46] Baldeon Calisto M Lai-Yuen SK. EMONAS-Net: efficient multiobjective neural architecture search using surrogate-assisted evolutionary algorithm for 3D medical image segmentation. Artif Intell Med. (2021) 119:102154. 10.1016/j.artmed.2021.102154 34531013

[47] Soerensen SJC Fan RE Seetharaman A Chen L Shao W Bhattacharya I Deep learning improves speed and accuracy of prostate gland segmentations on magnetic resonance imaging for targeted biopsy. J Urol. (2021) 206(3):604–12. 10.1097/ju.0000000000001783 33878887 PMC8352566

[48] Kouw WM Loog M. A review of domain adaptation without target labels. IEEE Trans Pattern Anal Mach Intell. (2021) 43(3):766–85. 10.1109/TPAMI.2019.2945942 31603771

[49] Montagne S Hamzaoui D Allera A Ezziane M Luzurier A Quint R Challenge of prostate MRI segmentation on T2-weighted images: inter-observer variability and impact of prostate morphology. Insights Imaging. (2021) 12(1):71. 10.1186/s13244-021-01010-9 34089410 PMC8179870

