# Supplementary material for: A multi-object deep neural network architecture to detect prostate anatomy in T2-weighted MRI: Performance evaluation
Source: Front Nucl Med. 2023 Feb 6;2:1083245. doi: 10.3389/fnume.2022.1083245 (PMC11460296; doi:10.3389/fnume.2022.1083245)
Supplement: Supplementary file 1 [file Datasheet1.docx]

**SUPPLEMENTAL SECTION for**

**Title: A Multi-object Deep Neural Network Architecture to detect Prostate Anatomy in T2-weighted MRI: Performance Evaluation**

Authors: Maria Baldeón Calisto^1,+^, Zhouping Wei^2,+^, Shatha Abudalou^2,3^, Yasin Yilmaz^3^, Kenneth Gage^4^, Julio Pow-Sang^5^, Yoganand Balagurunathan^2,*^.

^1^Universidad San Francisco de Quito, Quito, Ecuador

^2^Department of Machine Learning, ^4^Diagnostic Radiology, ^5^Genitourinary Oncology, Moffitt Cancer Center, Tampa, Florida, USA

^3^University of South Florida, Tampa, Florida, USA

*^+ equal contribution^*

*corresponding Author

Yoganand Balagurunathan, PhD

Department of Machine Learning,

H. Lee Moffitt Cancer Center and Research Institute

Tampa, FL 33612, USA

[yoganand.balagurunathan@moffitt.org](mailto:yoganand.balagurunathan@moffitt.org)

**Suppl. Table 1.** Prostate Gland volume and Dice Coefficients across quartiles based on gland volume.

| **Name index** | **Gland boundary**  **Delineation** | **Dice Coefficient**  *(Mean,95% Confidence, Median)* | | **Prostate Volume** (cm^3^),  *(Mean ,95% Confidence, Median)* | |
| --- | --- | --- | --- | --- | --- |
|  |  | **All Samples** | **Quartiles** | **All Samples** | **Quartiles** |
| **Train#1** | Consensus Radiology read (MCC) | n/a | n/a | 60.4,  [54.85,65.99],  53.6 | Q1:27.5,[25.93,29.07],27.6  Q2:44.5,[42.7,46.29],44.5  Q3:64.1,[61.93,66.2],64.3  Q4:106.4,[94.65,118.17],98.83 |
| Test#1 | Consensus Radiology read (MCC) | 0.854,  [0.843,0.868],  0.887 | Q1: 0.872,[.858,0.887],0.886  Q2: 0.858,[0.83,0.887],0.895  Q3: 0.820,[0.787,0.854],0.845  Q4: 0.870,[0.85,0.889],0.893 | 64.9,  [60.2, 69.6],  57.5 | Q1: 31.12, [29.3, 32.0], 31.7  Q2: 49.1, [47.9, 50.4], 49.1  Q3: 67.3, [65.4, 69.2], 66  Q4: 112.1, [104.8, 119.5],109.9 |
| Test#2 |  | 0.794,  [0.749,0.839],  0.805 | Q1:0.702,[0.573,0.831],0.71  Q2:0.798,[0.707,0.889],0.78  Q3:0.808,[0.762,0.854],0.803  Q4:0.883,[0.799,0.966],0.916 | 34.9,  [28, 41.8],  30.7 | Q1:23.6, [19.3, 28], 24.8  Q2:29.5, [28.7, 30.3],29.6  Q3:32.8, [31.2, 34.3],33.1  Q4:55.8,[28.4, 83.3],41.8 |
| Test#3 |  | 0.806,  [0.733,0.88],  0.842 | Q1:0.717,[0.367,1.07]  Q2:0.824,[0.63,1.02],0.875  Q3:0.864,[0.773,0.955],0.857  Q4:0.834,[0.747,0.92],0.828 | 44.2,  [ 27.7,60.7],  29.2 | Q1:21.7,[17.3, 26],22.8  Q2:25.2,[21, 29.4],23.9  Q3:42.9,[20.1, 65.6],46.5  Q4:86.8,[51.1, 122.5],95.5 |
| Test#4 | Organizers  Provided | 0.622,  [0.535,0.71],  0.737 | Q1:0.535,[0.314,-/757],0.73  Q2:0.435,[0.214,0.656],0.489  Q3:0.744,[0.664,0.824],0.811  Q4:0.77,[0.637,0.902],0.85 | 54.7,  [41.8, 67.6],  43.3 | Q1:28.2, [25, 31.6], 29.5  Q2:39, [37.1, 40.7], 39.1  Q3: 49, [46.8, 51.2], 48.5  Q4: 105.3, [59.5, 151.1],76.3 |

**Suppl. Table 2.** Distribution of PZ volume and Dice coefficients across quartiles based on PZ-volume.

| **Name index** | **PZ boundary** | **Dice Coefficient**  *(Mean,95% Confidence, Median)* | | **PZ Volume** (cm^3^),  *(Mean ,95% Confidence, Median)* | |
| --- | --- | --- | --- | --- | --- |
|  |  | **All Samples** | **Quartiles** | **All Samples** | **Quartiles** |
| **Train#1** | Consensus Radiology read (MCC) | n/a | n/a | 16.2,  [15.1,17.3],  14.3 | Q1:17.5,[ 15.1,20.0],15.9  Q2: 16.4,[ 13.9,18.9],13.8  Q3:14.5,[ 12.4,16.6],12.3  Q4:16.4,[ 14.2,18.5],14.4 |
| Test#1 | Consensus Radiology read (MCC) | 0.664,  [0.641,0.688],  0.718 | Q1:0.622,[0.565, 0.677],0.714  Q2:0.694,[0.651, 0.736], 0.73  Q3: 0.771,[ 0.749, 0.794],0.777  Q4: 0.57,[ 0.529, 0.611],0.58 | 18.2,  [16.7,19.6],  15.9 | Q1:23.1,[18.8,27.3],18.9  Q2:14.9,[13.1,16.8],14.5  Q3:13.8,[12.0,15.5],13.0  Q4: 20.9,[18.6,23.2],21.1 |
| Test#2 |  | 0.645,  [0.563,0.727],  0.686 | Q1:0.735,[ 0.64,0.831],0.685  Q2: 0.612,[ 0.346,0.879],0.744  Q3: 0.605,[ 0.38, 0.83],0.697  Q4: 0.623,[ 0.461,0.785],0.658 | 13.9,  [11.2,16.5],  13.5 | Q1:19.2,[13.1,25.3],16.5  Q2:13.1,[ 5.8, 20.5],9.3  Q3:13.2,[9.5,16.9],13.9  Q4:9.35,[6.5,12.2],9.82 |
| Test#3 |  | 0.640,  [0.457,0.717], 0.68 | Q1: 0.55,[ 0.0983,1.002],0.673  Q2: 0.659,[ 0.434,0.884],0.704  Q3: 0.609,[ 0.244,0.974],0.675  Q4: 0.53,[ -0.0436,1.1048],0.654 | 9.24,  [ 7.3,11.1],  10.2 | Q1:9.56,[6.55,12.5],9.69  Q2:9.08,[4.25,13.9],9.03  Q3:8.75,[1.45,16.05],8.96  Q4:9.67,[-3.64,22.9],11.7 |
| Test#4 | n/a | n/a | n/a | n/a | n/a |

**n/a: not available. No reference PZ region was available.*

**Suppl. Figure 1.** Segmentation performance evaluated across different gland volumes ordered by quartiles on estimated volumes (in cm^3^), in the test cohort. A) Test#1, B) Test#2, C) Test#3, D) Test#4.

**
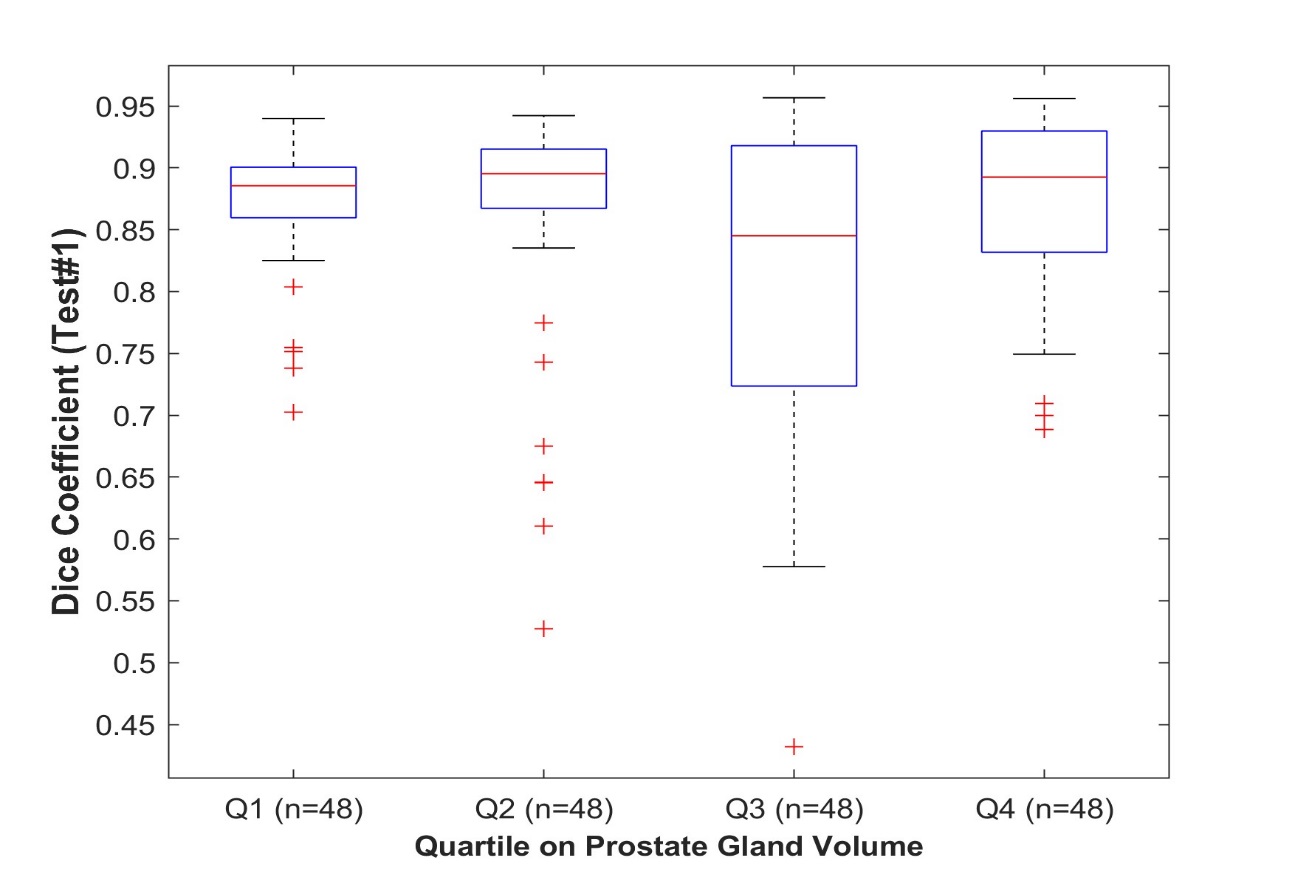
(A)
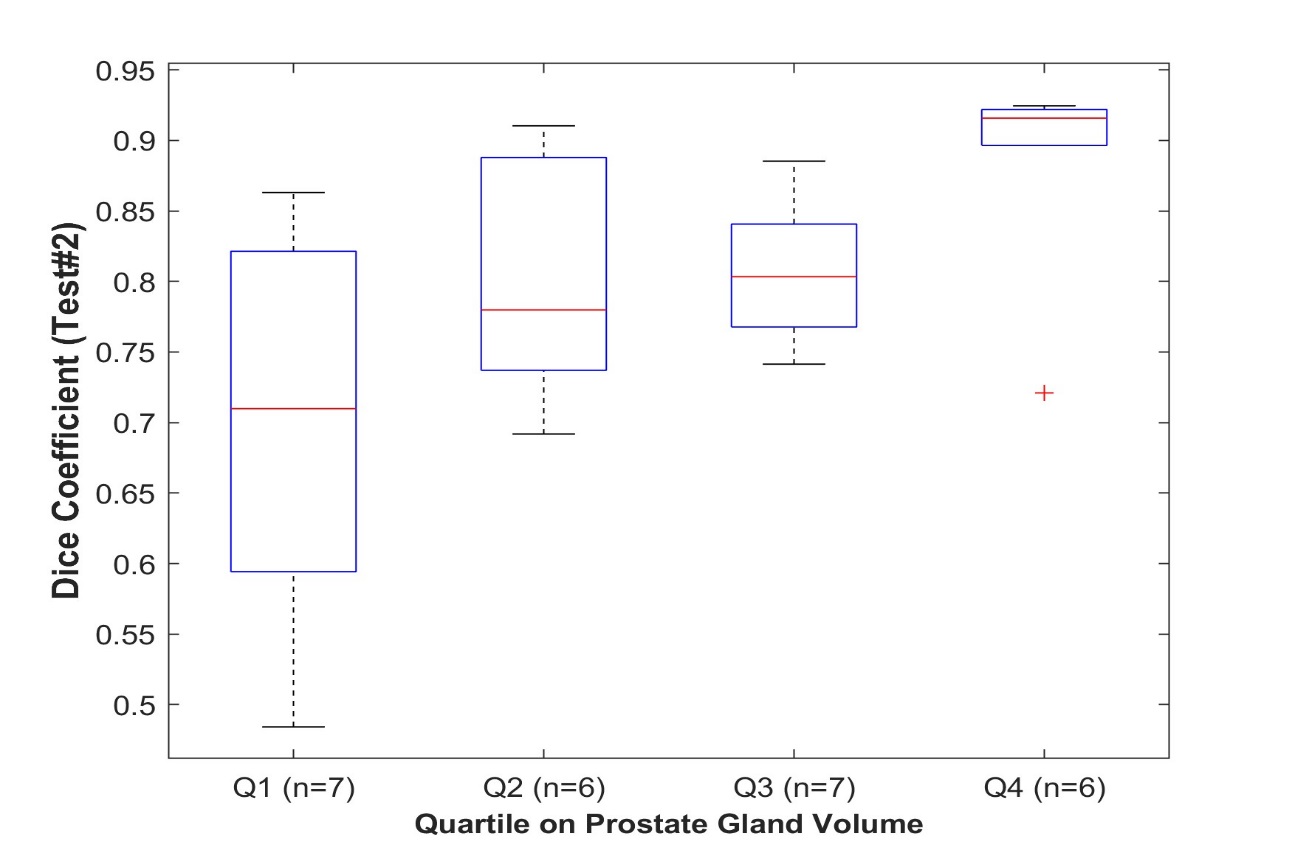
(B)**

**
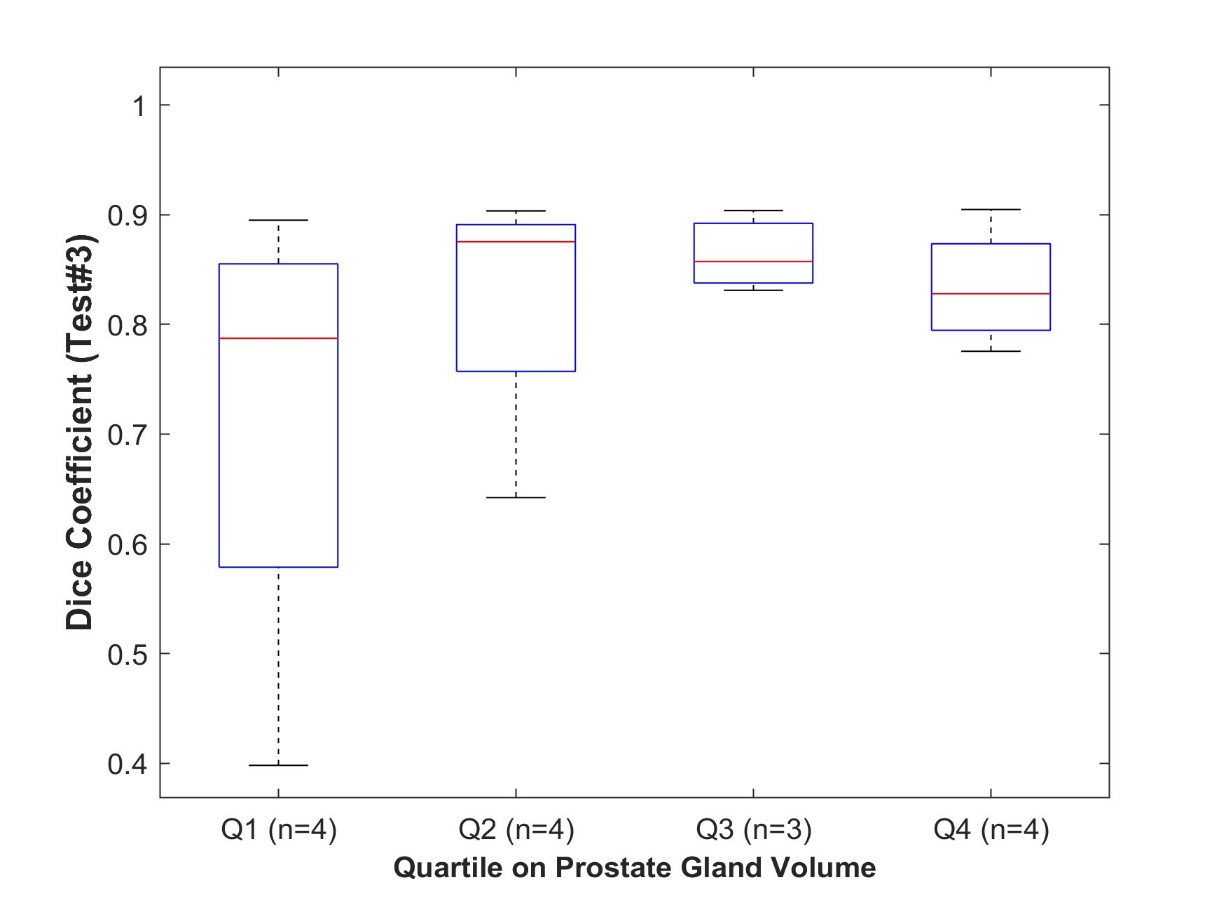
(C)**

**
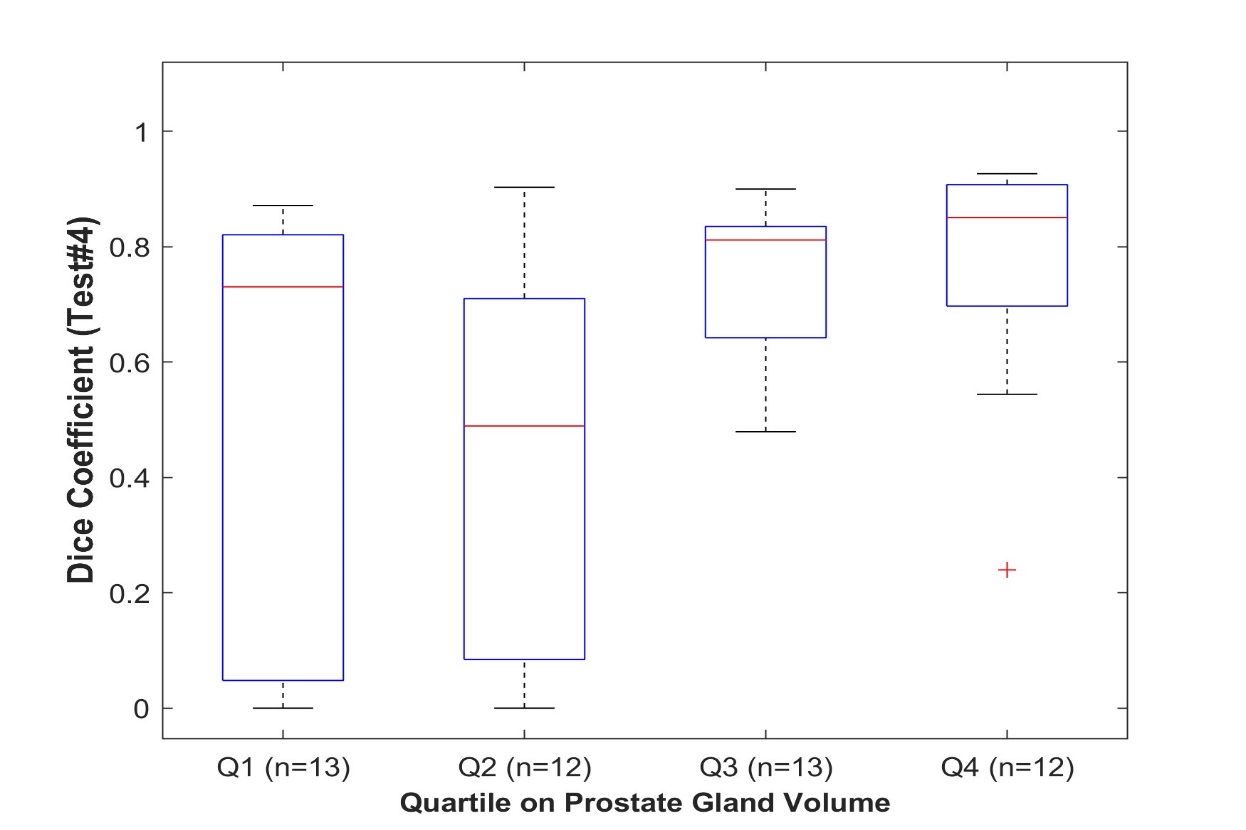
(D)**

**Suppl. Figure 2**. Segmentation performance evaluated across different PZ volumes ordered by quartiles on estimated volumes (in cm^3^), in the test cohort. A) All cohorts, B) Test#1, C) Test#2, D) Test#3. The Test#4 cohort did not have a PZ regional annotation.


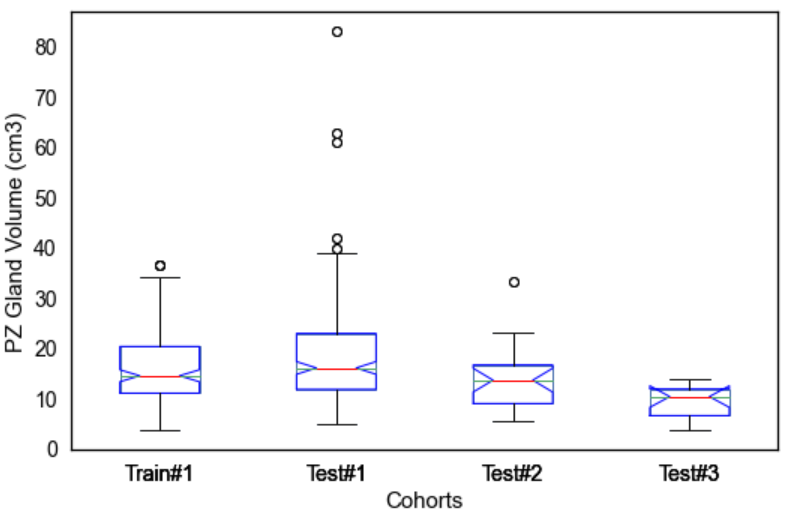
 **(A)**


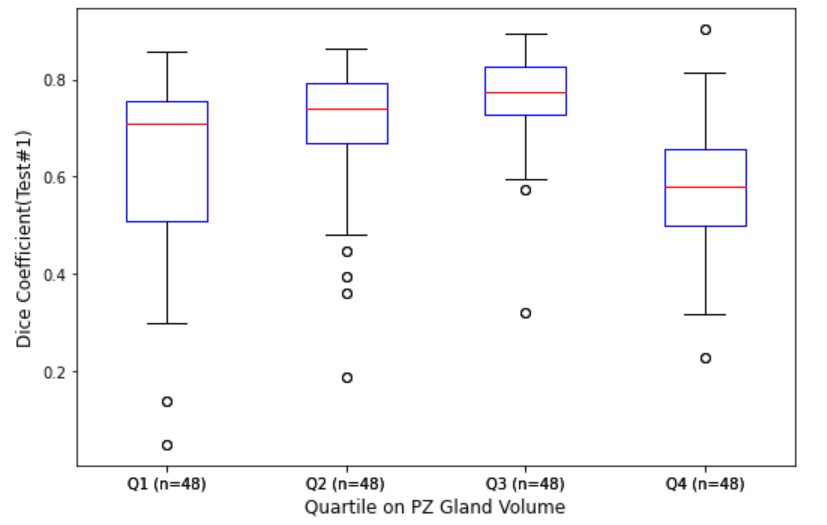
 **(B)**


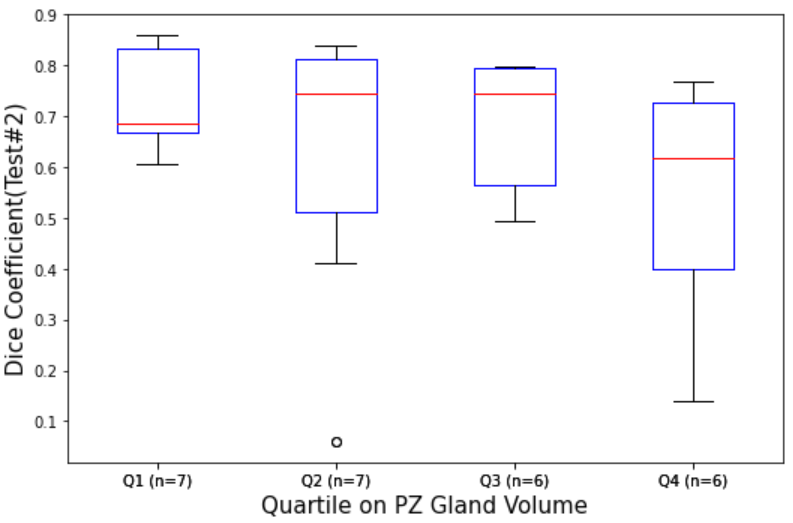
 **(C)**


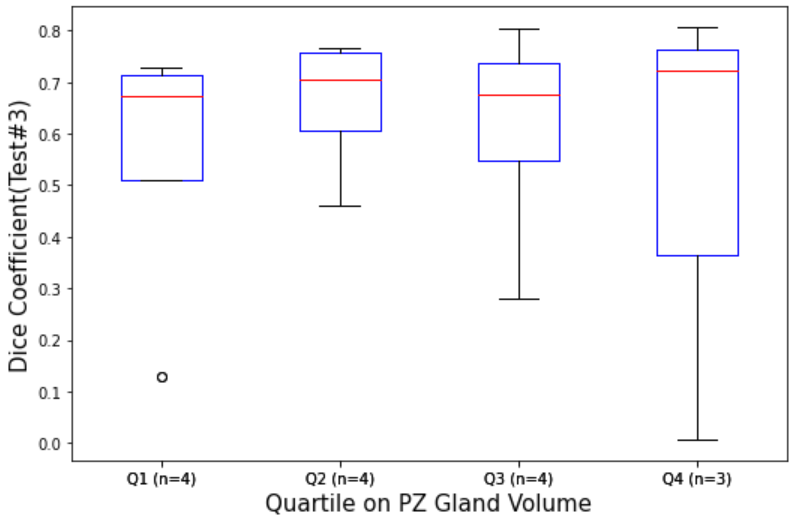
 **(D)**

**Suppl. Figure 3**. Scatter plot between estimated prostate gland volume with the segmentation performance statistics (Dice coefficient), across the test cohort. A) Test#1, B) Test#2, C) Test#3, D) Test#4.

**
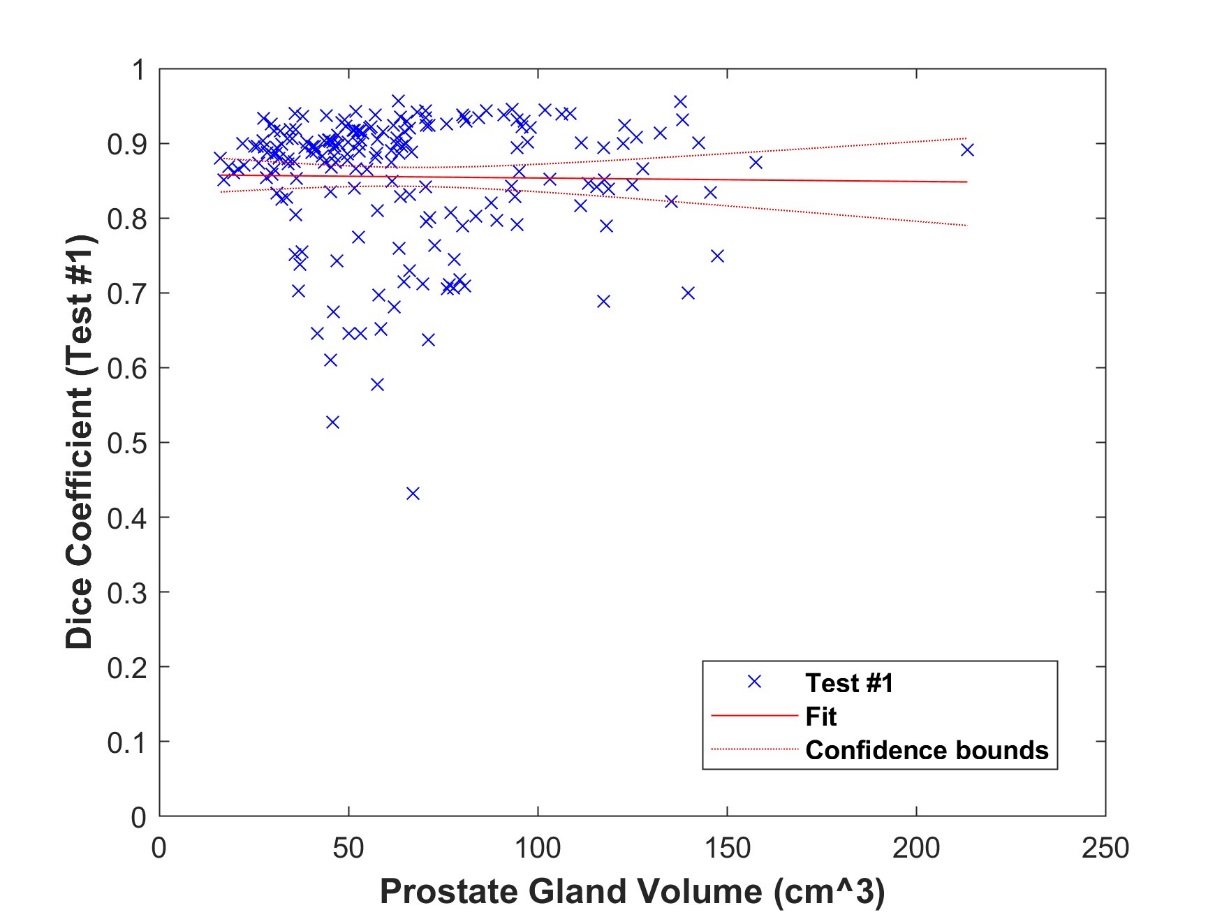
 (A)**

**
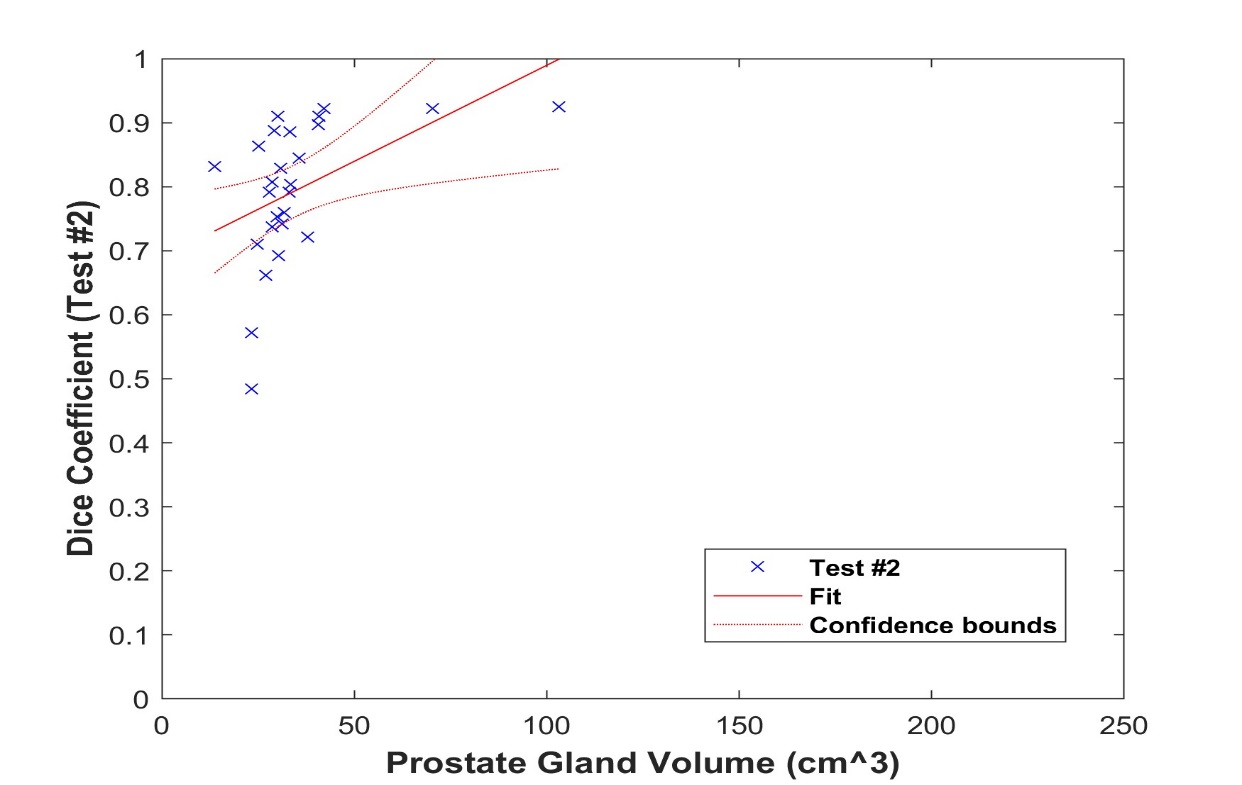
 (B)**

**
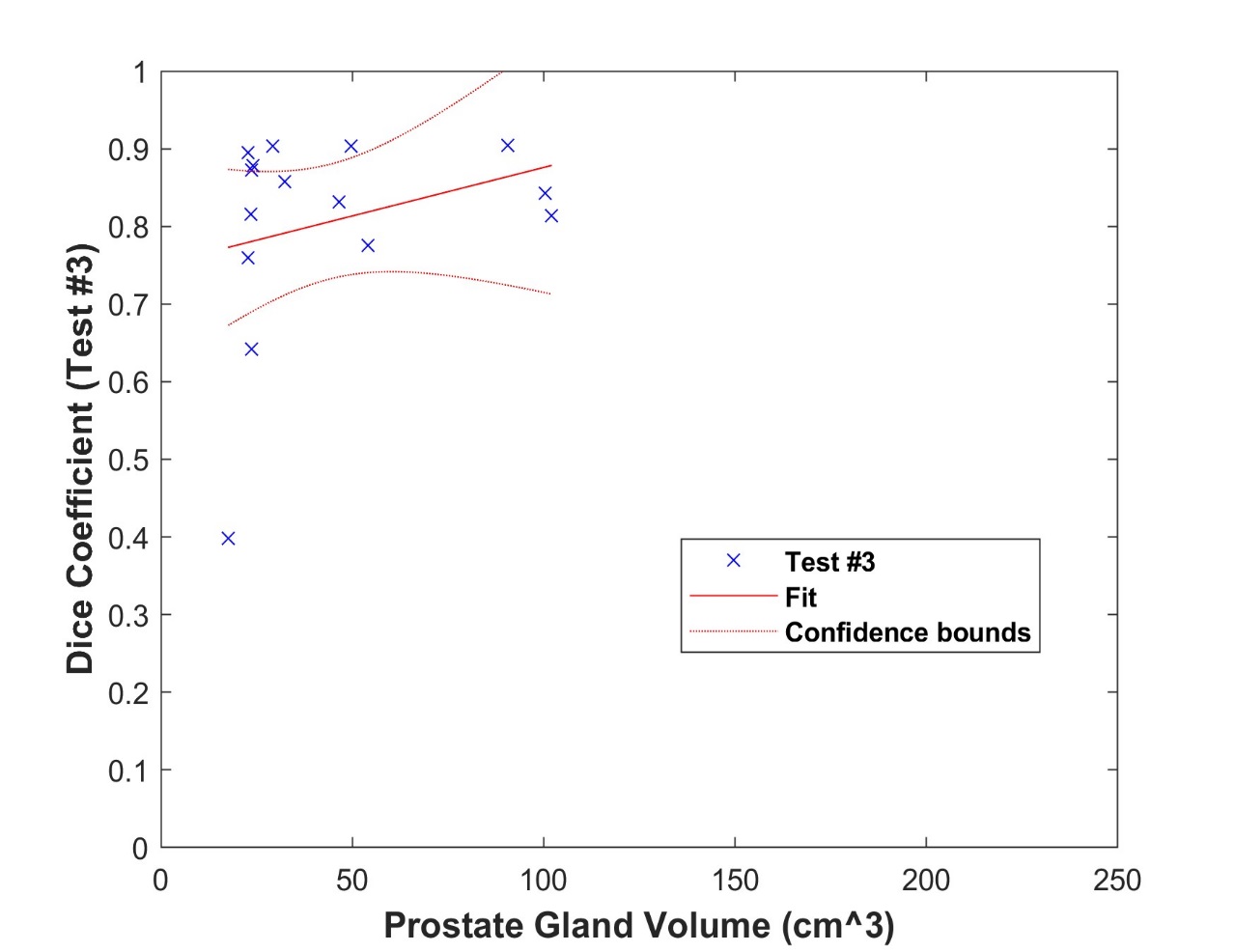
 (C)**

**
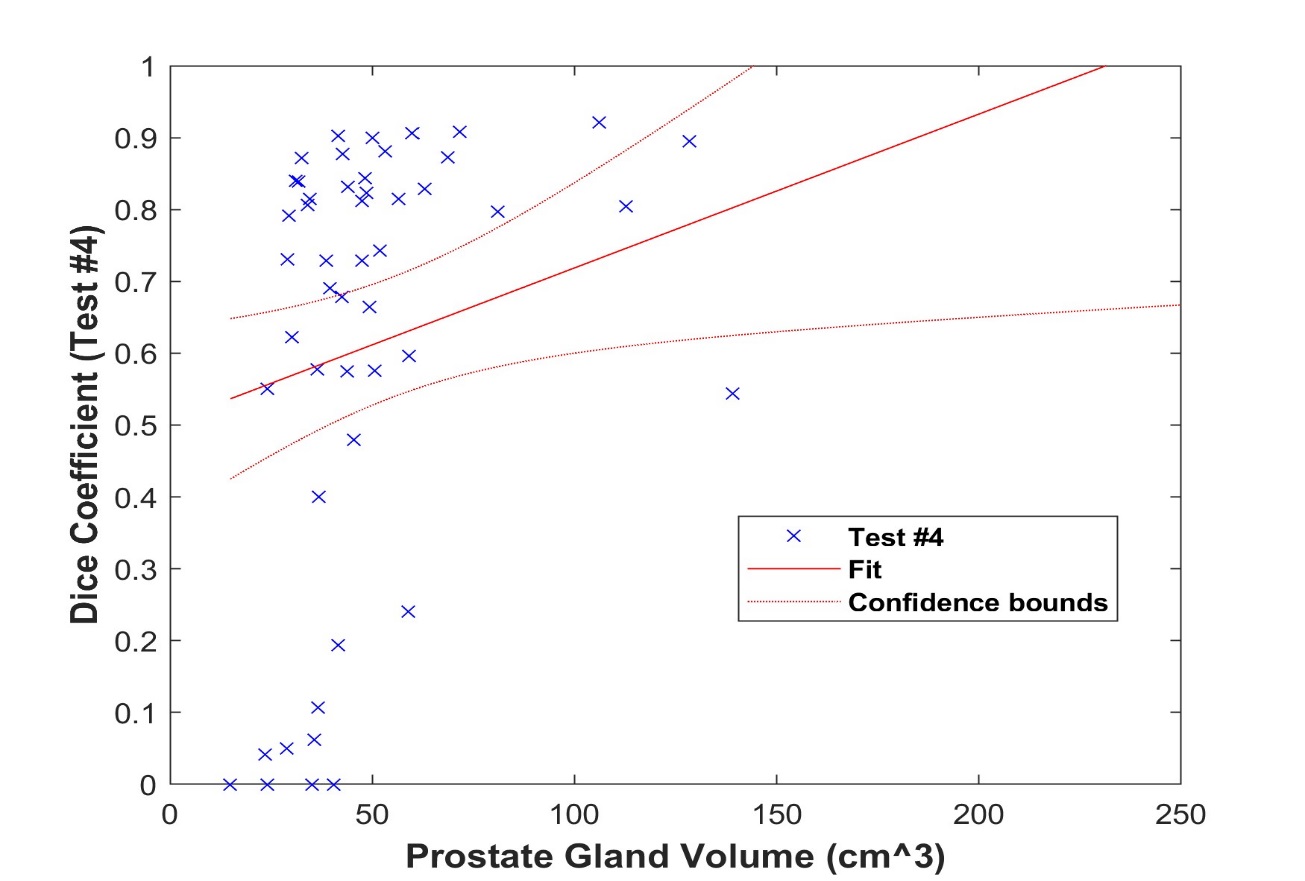
 (D)**

**Suppl. Figure 4**. Volume estimates for different range of Dice performance metrics ordered by quartiles. A) Test#1, B) Test#2, C) Test#3, D) Test#4.

**
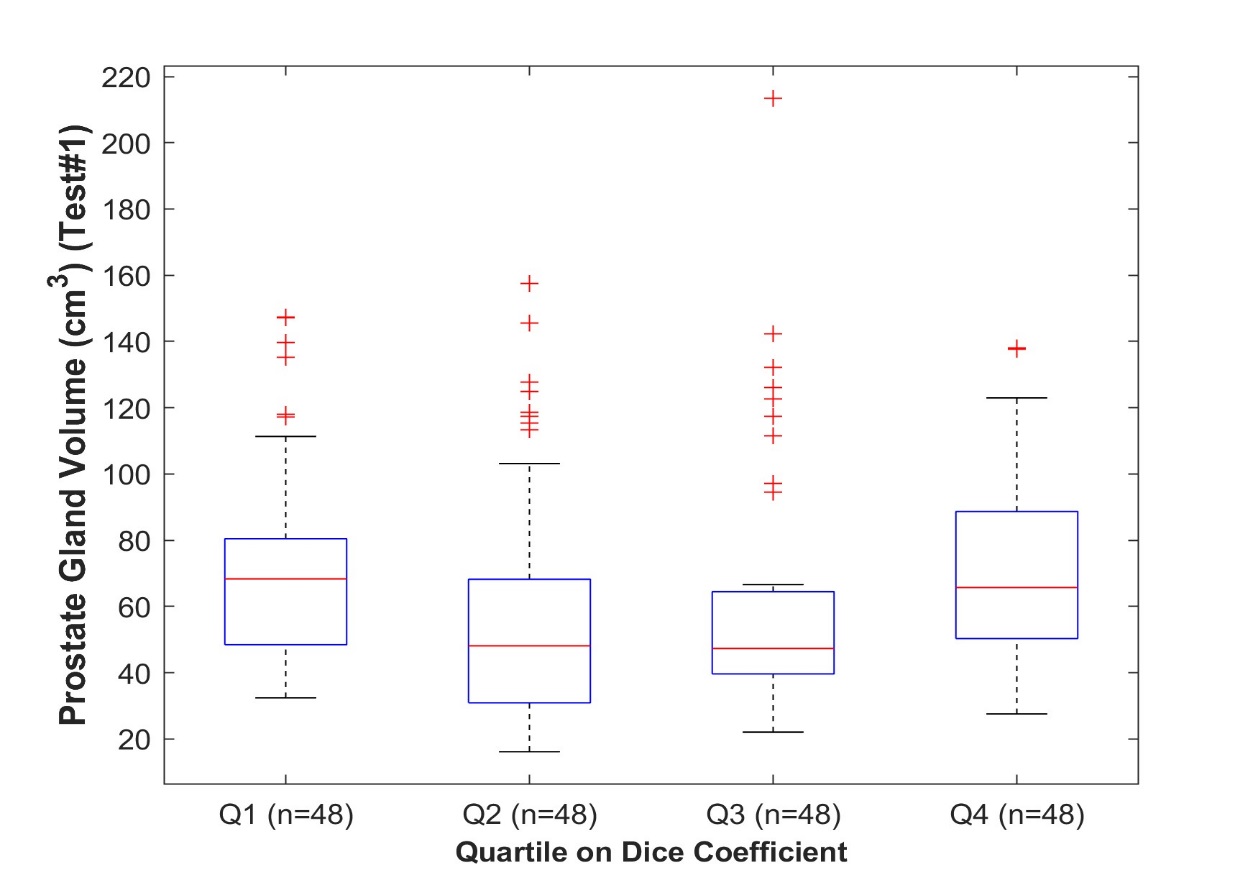
(A)**

**
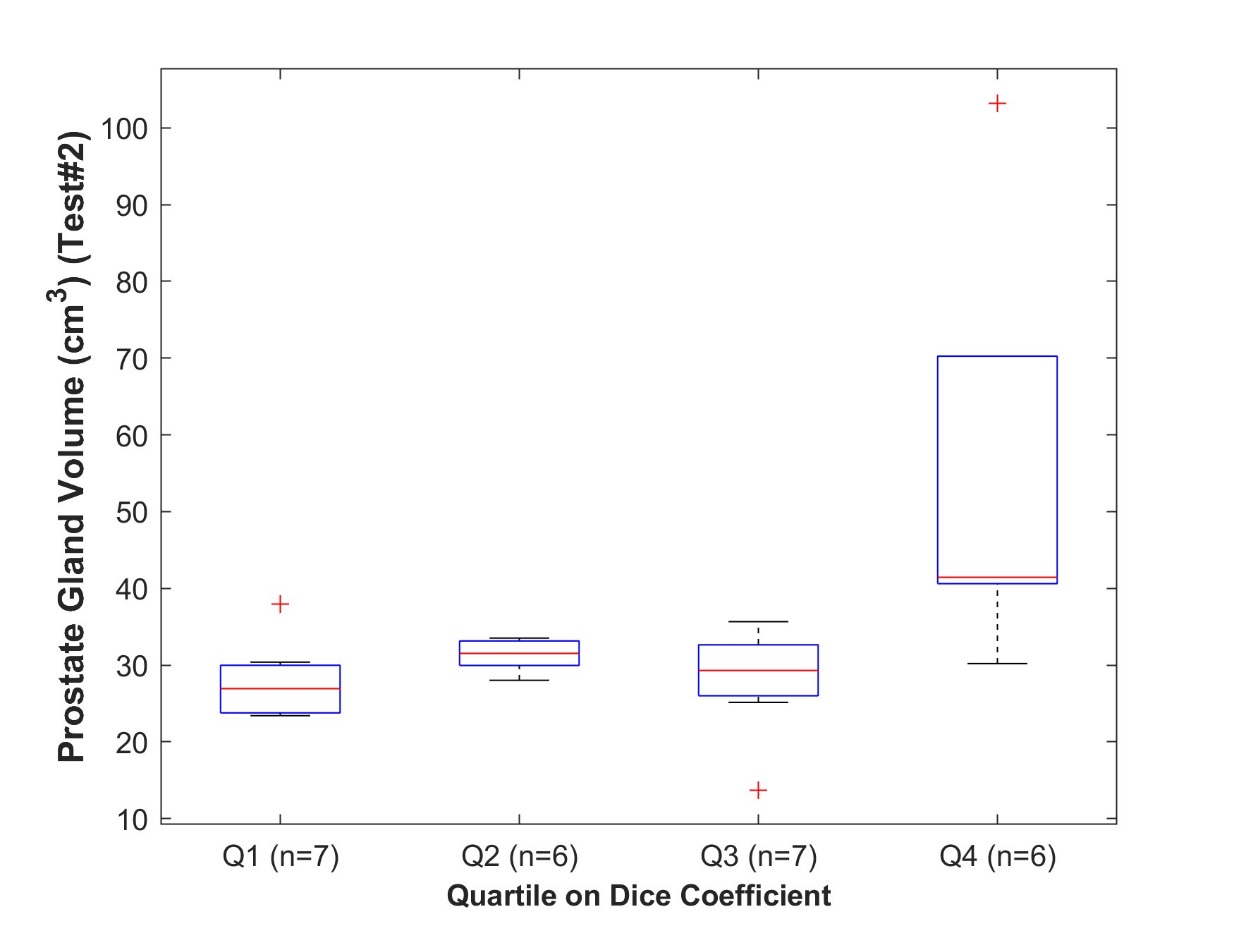
 (B)**

**
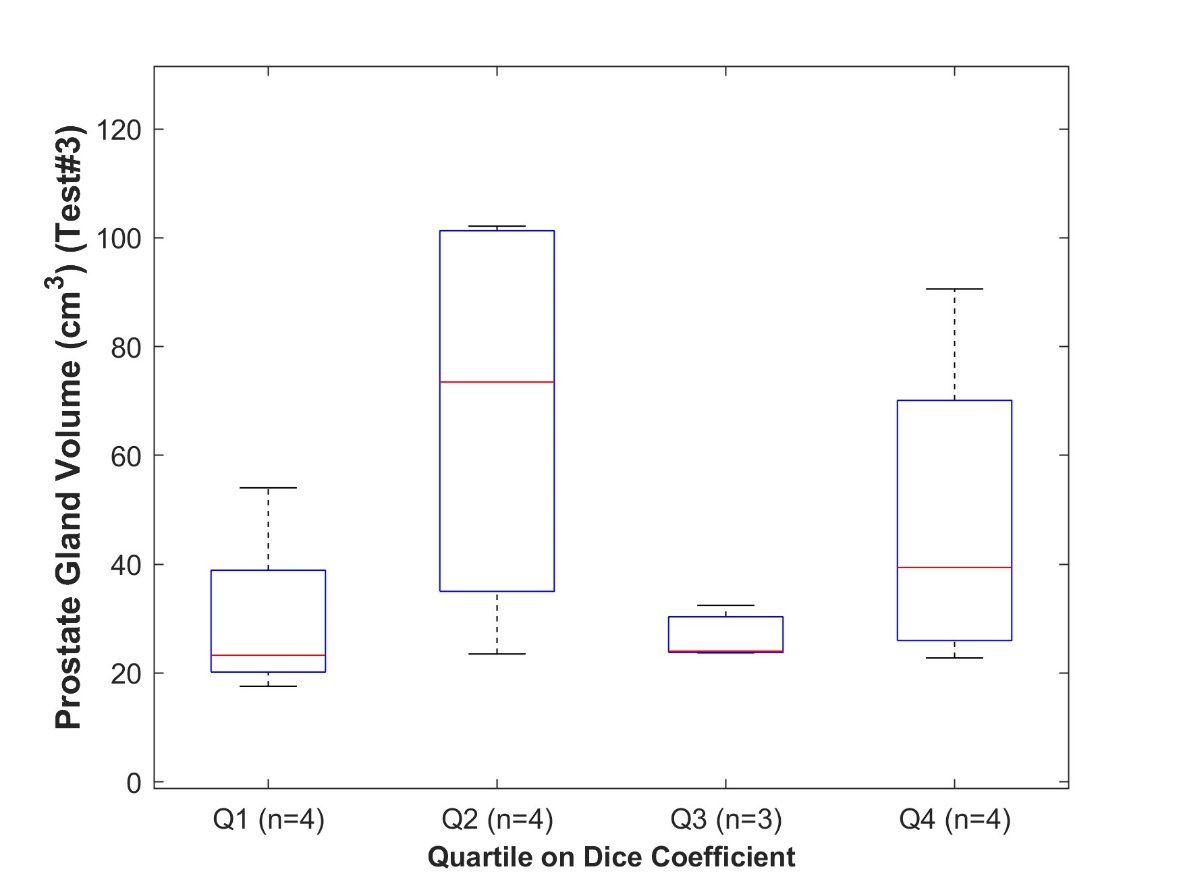
 (C)**

**
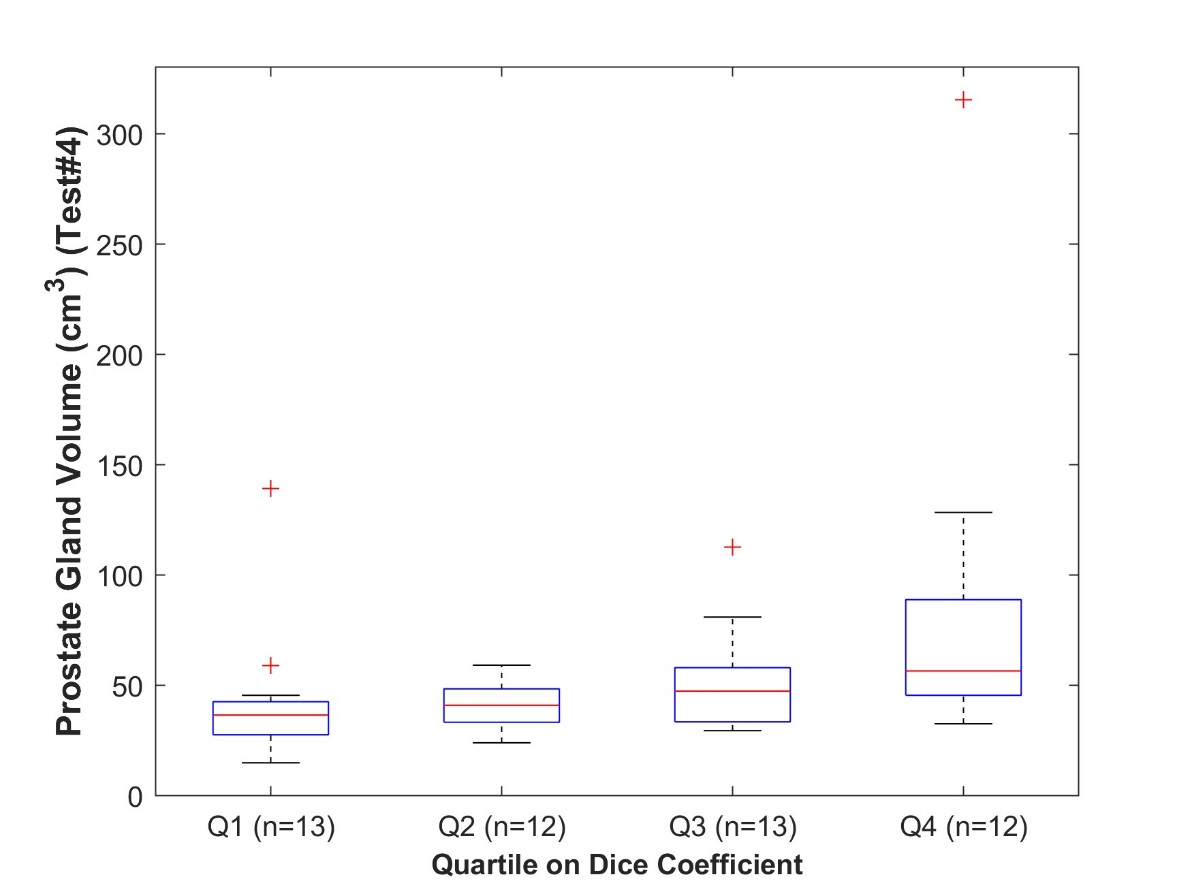
 (D)**
